# Genomic adaptation of *Clostridium perfringens* to human intestine

**DOI:** 10.1002/imo2.38

**Published:** 2024-10-25

**Authors:** Ke Wu, Juan Wang, Zelin Yan, Yanyan Zhu, Shaolin Wang, Bo Fu, Chengtao Sun, Ruichao Li, Edward M. Fox, Séamus Fanning, Li Bai, Yang Wang, Yizhi Tang, Zhe Yin, Rong Zhang, Hongning Wang

**Affiliations:** ^1^ Animal Disease Prevention and Food Safety Key Laboratory of Sichuan Province Key Laboratory of Bio‐Resource and Eco‐Environment of Ministry of Education, College of Life Sciences, Sichuan University Chengdu China; ^2^ Department of Preventive Veterinary Medicine, College of Veterinary Medicine Northwest A&F University Yangling China; ^3^ Research Unit of Food Safety Chinese Academy of Medical Sciences, NHC Key Lab of Food Safety Risk Assessment, China National Center for Food Safety Risk Assessment (CFSA) Beijing China; ^4^ Department of Clinical Laboratory Second Affiliated Hospital of Zhejiang University, School of Medicine Hangzhou China; ^5^ National Key Laboratory of Veterinary Public Health and Safety, College of Veterinary Medicine China Agricultural University Beijing China; ^6^ College of Veterinary Medicine Yangzhou University Yangzhou Jiangsu China; ^7^ Department of Applied Sciences Northumbria University Newcastle upon Tyne UK; ^8^ UCD‐Centre for Food Safety, School of Public Health, Physiotherapy and Sports Science University College Dublin Belfield Dublin Ireland; ^9^ State Key Laboratory of Pathogen and Biosecurity Academy of Military Medical Science Beijing China

**Keywords:** *Clostridium perfringens*, host relevance, population structure, virulence factor

## Abstract

*Clostridium perfringens* is often associated with foodborne diseases, posing significant public health risks. However, genomic investigation of *C. perfringens* isolates from the human population has been lacking. This study aims to fill this knowledge gap by examining the genomic characteristics of *C. perfringens* isolates from 699 individuals at a provincial hospital in China. We further conducted evolutionary and pan‐genomic analyses, incorporating isolates from humans and animals worldwide. The results reveal potential regional and transregional transmission of *C. perfringens* among individuals, along with the common transfer of small gene clusters during this process. Notably, the food poisoning‐associated toxin gene *cpe* was identified in a fusion plasmid for the first time in an isolate, indicating fusion of pCP13‐like and pCW3‐like plasmids and the potential for transfer of *cpe* across genetic backgrounds. Moreover, we observed that the genomic characteristics of *C. perfringens* correlate with host species, with specific toxin genes, such as *pfoA* and *colA*, potentially influencing host selectivity. Through this comprehensive genomic analysis, we provide novel insights into the fusion of pCW3‐like and pCP13‐like plasmids, the genetic location of *cpe*, the transmission dynamics of *C. perfringens* strains, and the relationship between toxin genes and host relevance. These findings expand our understanding of *C. perfringens* and its implications for public health.

## Highlights

- This study performed genomic analysis on *C. perfringens* from humans in China.
- There were regional and transregional transmissions of *C. perfringens* between individuals.
- *cpe* was first identified on a pCW3‐like and pCP13‐like fusion plasmid.
- Genomic characteristics of *C. perfringens* were associated with host species.

## Graphical abstract

This study performed genomic analysis on *Clostridium perfringens* from humans in China. There are regional and transregional transmissions of *C. perfringens* among individuals. *cpe* was first identified on a pCW3‐like and pCP13‐like fusion plasmid. Genomic characteristics of *C. perfringens* are associated with host species.

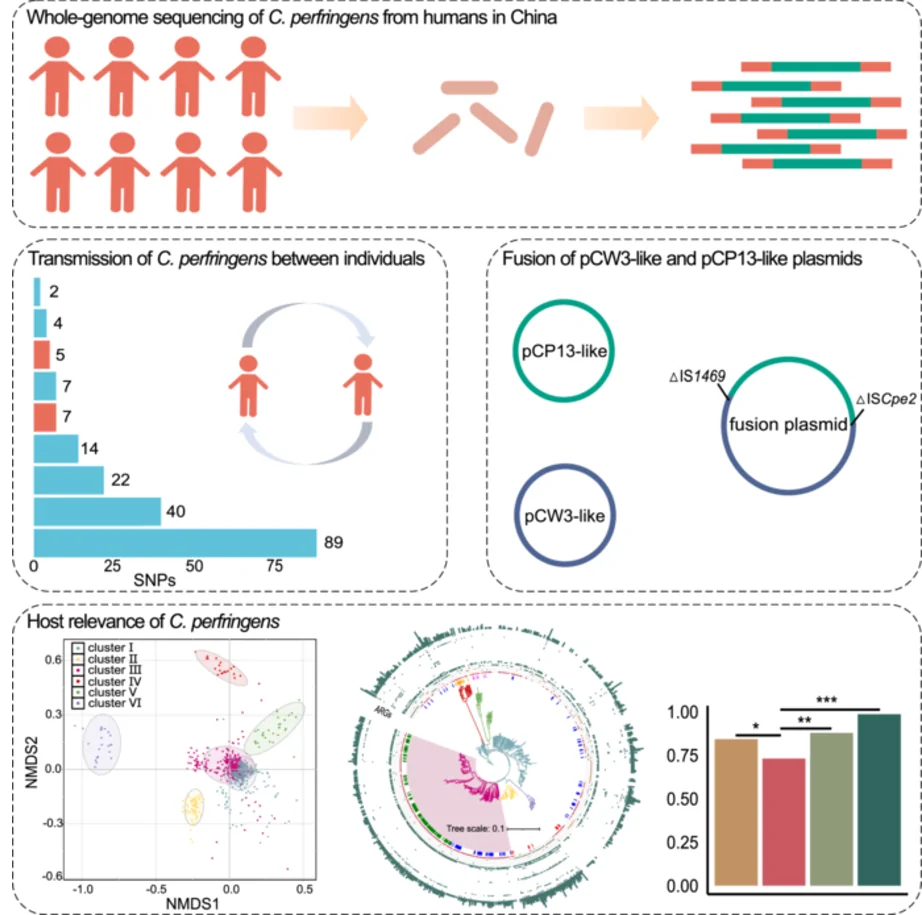

## 1 INTRODUCTION

*Clostridium perfringens* is one of the main foodborne pathogens responsible for diverse intestinal diseases in both humans and animals. Its pathogenicity is primarily attributed to the production of protein toxins and enzymes. Over 20 toxins have been identified from *C. perfringens*, causing a spectrum of diseases from histotoxic infections, such as gas gangrene, to intestinal infections, including enteritis [1, 2]. *C. perfringens* is classified into seven toxin types (A–G) according to the production of six major toxins: α‐toxin (*plc*), β‐toxin (*cpb*), ε‐toxin (*etx*), ι‐toxin (*iap/ibp*), *C. perfringens* enterotoxin (*cpe*), and necrotic enteritis B‐like toxin (*netB*) [3].

*C. perfringens* is widely distributed in the environment and foods. Its heat‐resistant spores can germinate and proliferate in undercooked foods, leading to potential infections [4]. After ingestion, the pathogenic strains rapidly proliferate and secrete large amounts of protein toxins, causing intestinal infection and food poisoning. *C. perfringens* has been associated with conditions such as antibiotic‐associated diarrhea (AAD) and necrotizing enterocolitis in humans [5], and has been implicated in numerous foodborne outbreaks worldwide [6, 7, 8], posing a significant public health threat.

Plasmids play an important role in the virulence of *Clostridial* species. Based on the conjugative loci, plasmids of *C. perfringens* are divided into three main categories, the conjugative pCW3‐like plasmids (Tcp locus), pCP3‐like plasmids (Pcp locus), and the non‐conjugative pIP404‐like plasmids [9, 10]. All of the major toxin genes, except for *plc*, are carried by pCW3‐like plasmids; similarly, several antibiotic resistance genes (ARGs), such as *tetA/B*(P) and *erm*(Q), are associated with pCW3‐like plasmids [11, 12, 13, 14]. *C. perfringens* type F isolates that carry *cpe* on the chromosome or on the pCW3‐like plasmids are considered the major cause of foodborne infections. These isolates sporulate and produce CPE in the intestine of a susceptible host, causing food poisoning [15, 16]. A recent study reported that the binary enterotoxin BEC (encoded by *becA/B* on pCP13‐like plasmids) is also linked to foodborne gastroenteritis caused by *C. perfringens* [17, 18]. High‐throughput sequencing technology has been widely applied in epidemiological studies to facilitate the characterization of potential pathogen transmission routes [19, 20]. As a zoonotic foodborne pathogen, understanding the genomic characteristics of *C. perfringens* is crucial to public health. While some studies have investigated the genomes of *C. perfringens* from animals such as sheep and pigs in China [21, 22], there has been no research focusing on the population genomic characteristics of human‐derived *C. perfringens* in the country to date.

In the present study, we describe a comprehensive genomic analysis of *C. perfringens* isolates from the intestinal tracts of humans in China. We have identified key genetic markers, including ARGs, toxin genes, and plasmids, and traced their putative transmissions among individuals. Additionally, comparative studies between the *C. perfringens* isolates and those available from humans and animals worldwide were conducted to gain insight into the population structure and host tropism of *C. perfringens*.

## 2 RESULTS

### 2.1 Genetic marker of the *C. perfringens* strains

**Figure 1:**
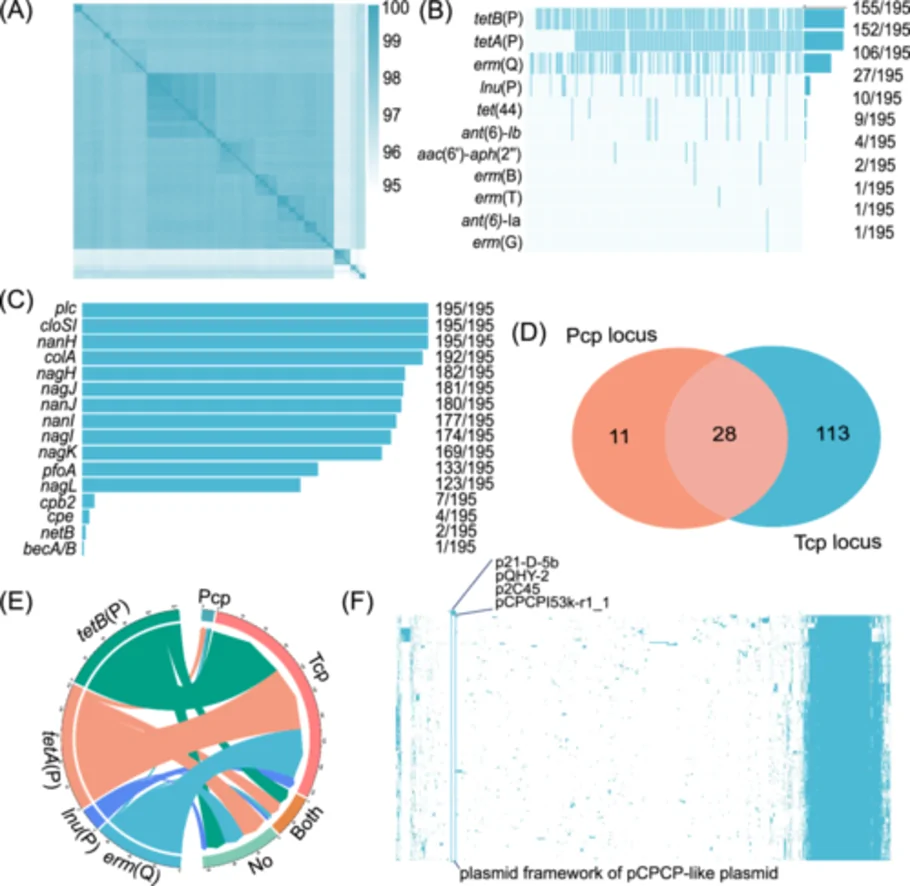
Genomic information of the 195 *Clostridium perfringens* isolates from humans in this study. (A) Average nucleotide identity (ANI) between the isolates. (B) Antibiotic resistance genes (ARGs) of the *C. perfringens* isolates. (C) Prevalence of toxin genes among the *C. perfringens* isolates. (D) Identification of Pcp and Tcp loci among the isolates. (E) Link between ARGs and Tcp/Pcp locus. (F) Detection of the plasmid framework of the novel pCPCP‐like plasmids among the isolates. Plasmid framework of pCPCP‐like plasmids is marked by box.

A total of 195 *C. perfringens* genomes that passed quality control were included in the analysis (Table S1). The average nucleotide identity (ANI) among these genomes ranged from 95% to 100% (Figure 1A). Most of these isolates (190/195, 97.4%) were identified as *C. perfringens* type A, whereas four isolates (CQ145, CQ147, CQ165, and CQ169) were identified as type F.

ARGs representing four antibiotic classes were identified among the isolates (Figure 1B). These include tetracycline resistance genes (*tetA*(P), *tetB*(P), and *tet*(44)), macrolide resistance genes (*erm*(B), *erm*(G), *erm*(Q), and *erm*(T)), aminoglycoside resistance genes (*ant*(6)‐*Ia*, *ant*(6)‐*Ib*, and *aac*(6′)‐*aph*(2”)), and lincomycin resistance gene (*lnu*(P)). Additionally, 16 toxin‐encoding genes, including *cpe* and *becA/B*, were identified (Figure 1C).

The distribution of plasmids and ARGs among the isolates was also analyzed. The Tcp and Pcp loci coding genes were detected and shown in Table S2. A pan‐genomic comparison between plasmids pCW3, pCP13, and the *C. perfringens* isolates is provided in Table S3. The results showed that the pCP13‐like plasmids were identified in 20% (39/195) of the isolates, while the pCW3‐like plasmids were significantly more prevalent (141/195, 72.3%, *p* < 0.01) (Figure 1D). The major ARGs [*tetA*(P), *tetB*(P), *erm*(Q), and *lnu*(P)] were detected among the *C. perfringens* isolates carrying different plasmid profiles (Figure 1E). Notably, the novel pCPCP‐like plasmid type was not identified in the isolates (Figure 1F).

### 2.2 Transmission of *C. perfringens* strains

**Figure 2:**
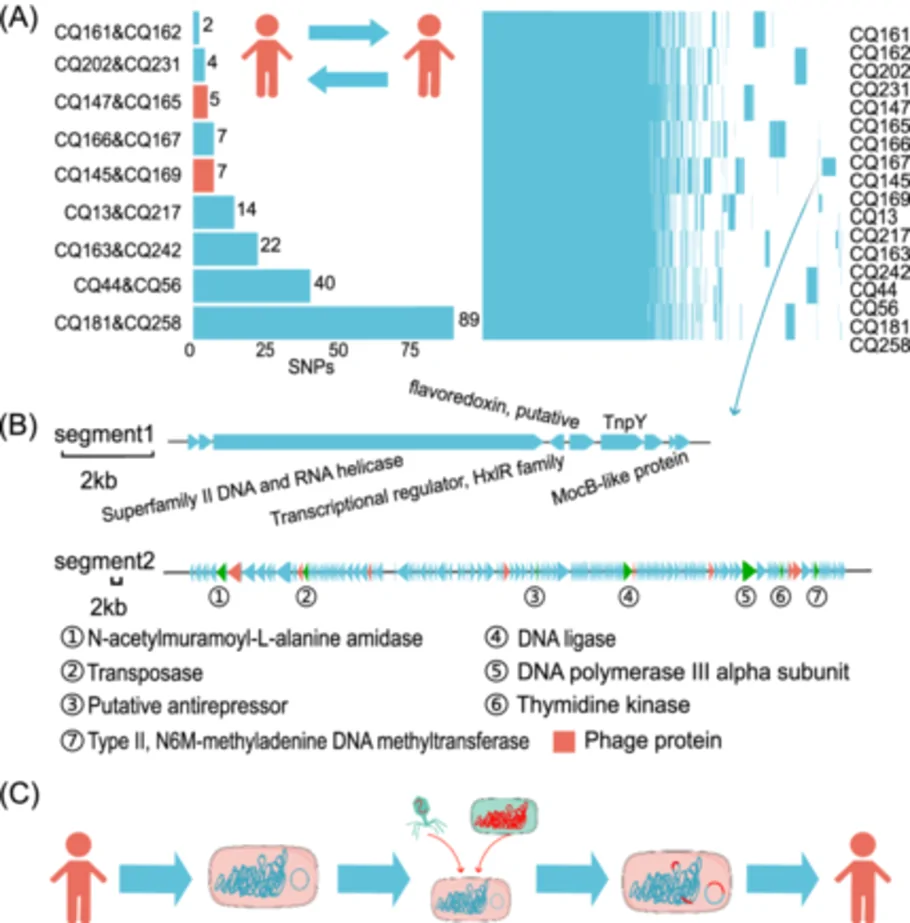
Potential transmission of *Clostridium perfringens* among humans. (A) Single nucleotide polymorphisms (SNPs) and pan‐genome of the isolates. (B) Extra sequence segments in *C. perfringens* isolate CQ145. (C) Schematic diagram of the potential transmission of *C. perfringens* strains. During this process, *C. perfringens* can acquire gene fragments from phages and other bacteria. In the diagram, *C. perfringens* is represented in pink, while phage and other bacteria are depicted in light turquoise.

The dissemination of pathogenic bacterial strains is a primary driver of foodborne disease outbreaks. An ANI analysis revealed that eight isolates (CQ161&CQ162, CQ202&CQ231, CQ147&CQ165, CQ166&CQ167, CQ145&CQ169, CQ13&CQ217, CQ163&CQ242, CQ44&CQ56, CQ181&CQ258) exhibited an average nucleotide identity of >99.99%. Furthermore, single‐nucleotide polymorphism (SNP) calling and pan‐genomic showed that these isolates shared SNP differences ranging from 2 to 89 and displayed a similar genomic composition (Figure 2A), suggesting a potential transmission event. Notably, a comparative genomic analysis of the *C. perfringens* strains CQ145 and CQ169 identified the presence of several additional genes in the genome of CQ145, which were located on two gene clusters designated as *segment 1* and *segment 2*.

Gene annotation characterized several functional proteins, including TnpY and a MocB‐like protein, were encoded by genes located on *segment 1*. TnpY and MocB are components of the transposons Tn*4451*/Tn*4453* and Tn*4399*, respectively [23, 24]. Tn*4451* and Tn*4453* have been associated with the transfer of ARGs in *C. perfringens* and *Clostridiodies difficile* [25]. A recent study indicates that Tn*4451*/Tn*4453* and Tn*As3* are attributed to ARG transfer between bacteria from human and food‐producing animal sources [26]. In addition, BLAST analysis showed similar sequences in various other bacterial species, including *Enterococcus faecalis* (Table S4), suggesting the potential for horizontal transfer of *segment 1* among diverse bacterial genera. In contrast to *segment 1*, *segment 2* contained several genes encoding phage proteins associated with temperate bacteriophages (Figure 2B). These findings indicate that *C. perfringens* strains can acquire external gene clusters during transmission, which may enhance their ability to incorporate and disseminate new genes in subsequent transmission processes (Figure 2C).

### 2.3 Genetic environments of *cpe* and *becA/B*

**Figure 3:**
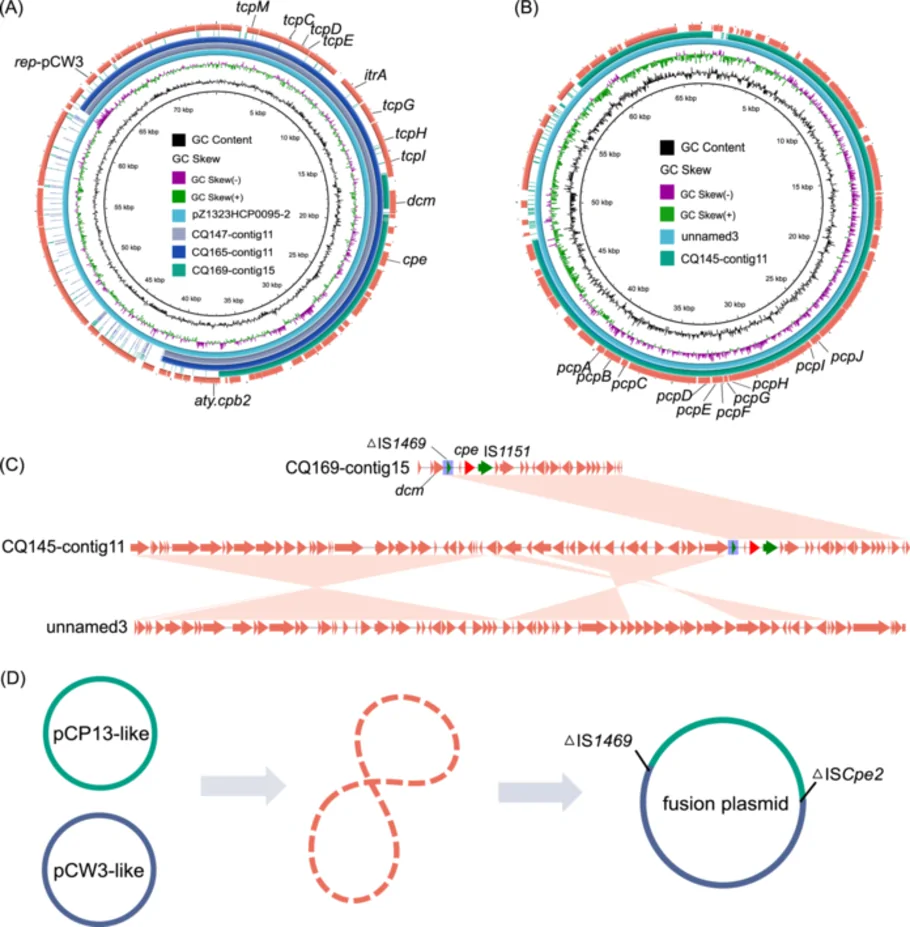
Genetic environments of *cpe* genes in *Clostridium perfringens* type F strains CQ145, CQ147, CQ165, and CQ169. (A) Sequence comparison of the pCW3‐like plasmid pZ1323HCP0095‐2 and the *cpe*‐positive contigs in CQ147, CQ165, and CQ169. (B) Circler comparison of pCP13‐like plasmid unnamed3 and the *cpe*‐positive contig in CQ145. (C) Structure of the *cpe*‐positive contig in CQ145. (D) Fusion plasmid formation schematic. Insertion sequences (ISs) △IS*1469* and △IS*Cpe2* lead to the fusion of pCW3‐like and pCP13‐like plasmids.

Understanding the genetic characteristics of virulence factors associated with clinical illness is crucial for controlling foodborne diseases. The *cpe* gene in CQ147 and CQ167 was found on a contig containing the *tcp* locus, and shared high sequence identity with the pCW3‐like plasmid pZ1323HCP0095‐2 (NZ_CP148594) (Figure 3A). In contrast, the *tcp* locus was absent in the *cpe*‐positive contigs of CQ145 or CQ169.

Sequence annotation showed that the *cpe* gene was located alongside *dcm* in CQ169, flanked by △IS*1469* and IS*1151*. Interestingly, a Pcp locus was identified in the *cpe*‐positive contig in CQ145 (Figure 3B). Sequence analysis showed that this contig was composed of a part of the pCP13‐like plasmid unnamed3 (CP134265.1) and a partial sequence (starting with △IS*1469*) from the *cpe*‐positive contig of CQ169 (Figure 3C).

To rule out potential errors in the assembly process, we performed PCR/Sanger sequencing using primers targeting the junction region in the *cpe*‐positive contig of CQ145. The results were consistent with the WGS data generated by the Illumina HiSeq X Ten System (Figure S1). However, the initial WGS analysis did not retrieve the complete sequences of the plasmids CQ145. To further characterize these plasmids, we conducted WGS analysis of CQ145 using the PacBio platform. The results (BioProject: PRJNA1118504) revealed that the Pcp and Tcp loci were both located on plasmid pcq145‐tcppcp, along with *cpe*, suggesting the fusion of pCW3‐like and pCP13‐like plasmids in CQ145. Subsequent BLAST analysis of pcq145‐tcppcp on NCBI showed high sequence identity with the pCW3‐like plasmid pZ1323HCP0095‐2 (CP148594.1) and the pCP13‐like plasmid pZ1323HCP0095‐3 (CP148595.1). Sequence comparison indicated that pcq145‐tcppcp resulted from integration of the pCW3‐like plasmid into the collagen adhesin coding gene *cna* on the pCP13‐like plasmid. Notably, △IS*1469* and △IS*Cpe2* were located at the junctions between the pCW3‐like and pCP13‐like plasmids (Figures 3D and S2), suggesting that these elements played a role in the formation of pcq145‐tcppcp.

The *becA/B*‐positive contig12 in strain CQ92 was also found to contain the Pcp locus and shared a high sequence identity with similar loci previously identified on the pCP13‐like plasmid pCP‐TS1 (AP024974.1) (Figure S3).

### 2.4 Population structure of *C. perfringens* in China

**Figure 4:**
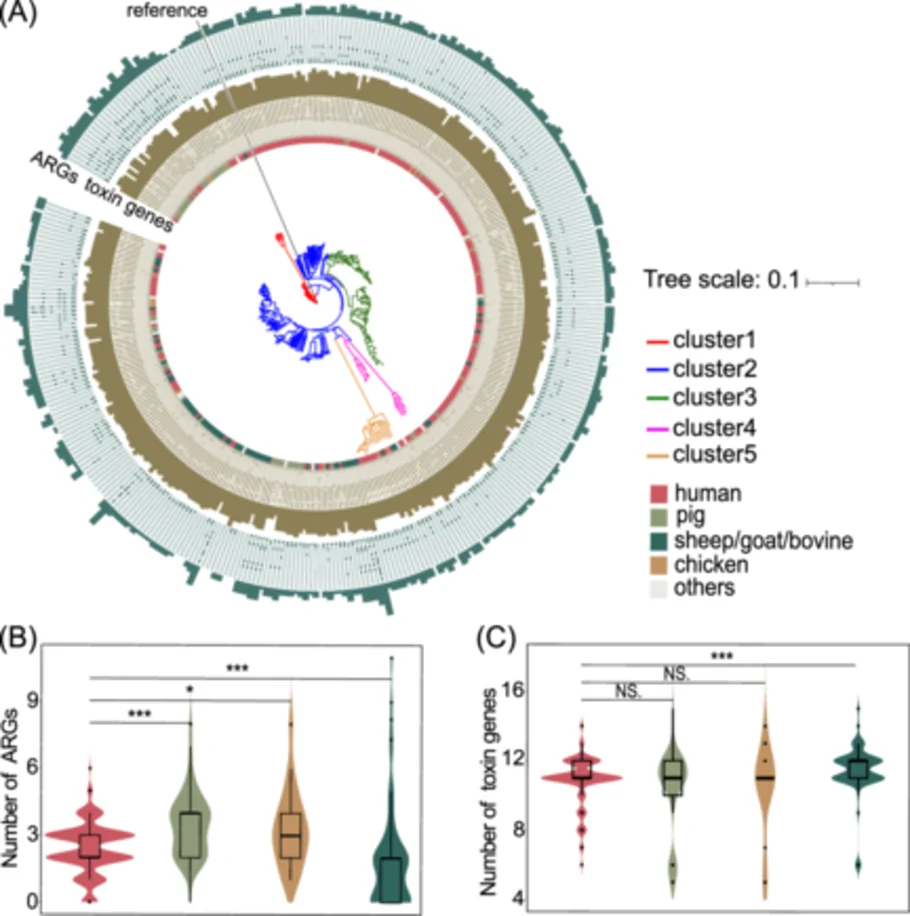
Phylogenetic analysis of *Clostridium perfringens* strains in China. (A) Maximum‐likelihood tree of the 195 *C. perfringens* isolates in this study and the other 167 *C. perfringens* strains available in China. (B) Comparison of the total number of antibiotic resistance genes (ARGs) and (C) toxin genes between humans and animal isolates.

To investigate the relationship between human and animal *C. perfringens* in China, we performed an evolutionary analysis based on 162,222 core‐genome SNPs from the 195 human‐derived isolates in this study and 167 *C. perfringens* strains available in China (Table S5). The maximum‐likelihood tree analysis revealed five evolutionary clades, denoted as clusters 1 through 5 (Figure 4A). The human‐derived strains clustered in clusters 2 and 3, while the animal‐derived strains clustered in clusters 1 and 2. Fewer human‐derived strains appeared in clusters 1 and 4, which mainly contain strains derived from animals, such as pig and chicken. In cluster 5, 11 human‐derived strains were observed to cluster with some strains from chicken and bovines.

Several clinically important ARGs, including *optrA*, cfr(B), and cfr(C), were exclusively detected in animal‐derived strains. A comparison of the total number of ARGs showed that animals, particularly pigs, were the main reservoirs of ARGs in China (Figure 4B). In addition, the sheep, goat, and bovine‐derived strains harbored more toxin genes than those from humans (Figure 4C), suggesting potential differences in virulence among strains from different animal sources.

### 2.5 Evolutionary relationship of *C. perfringens* from humans worldwide

**Figure 5:**
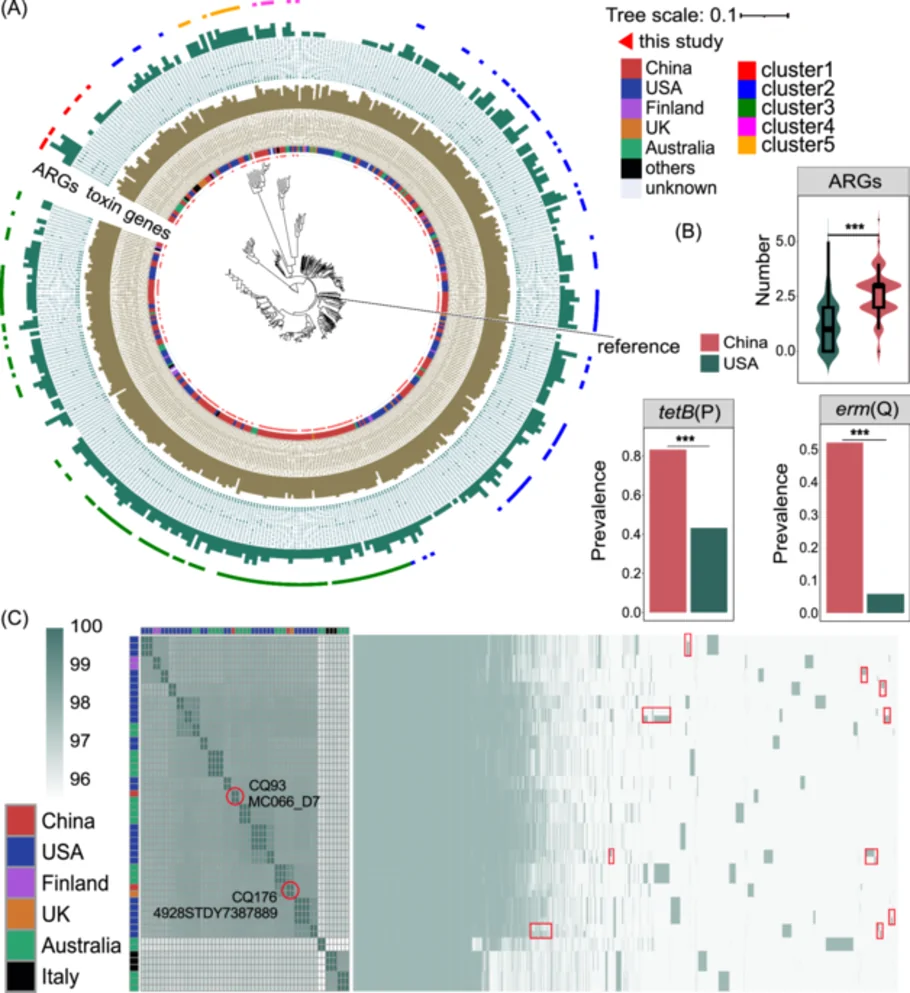
Evolutionary analysis of the 423 *Clostridium perfringens* strains from humans worldwide. (A) Maximum‐likelihood tree of the *C. perfringens* strains. (B) Comparison of the total number of antibiotic resistance genes (ARGs) and detection rates of *erm*(Q) and *tetB*(P) between the strains from China and the United States. (C) Genomic composition of the *C. perfringens* strains with high ANIs, small genetic differences are marked by red squares.

Herein, we investigated the genomic characteristics and phylogenetic relationships of 423 *C. perfringens* strains derived from humans worldwide, with detailed metadata provided in Table S6. The majority of the isolates were grouped closely in the maximum‐likelihood tree based on 118,750 core‐genome SNPs from the human‐derived strains worldwide (Figure 5A), showing highly similar ANIs, regardless of their country of origin (Figure S4A). Furthermore, no significant variances were observed in the accessory gene profiles of isolates from different countries (Figure S4B), suggesting that the regional sources did not significantly influence the evolutionary relationship or genomic characteristics of *C. perfringens*.

A total of 19 ARGs were identified among the human‐derived isolates, with *tetA*(P) (326/423, 77.1%), *tetB*(P) (252/423, 57.9%), and *erm*(Q) (121/423, 28.6%) being the most prevalent. In contrast, *lnu*(P) was identified in only 8.0% (34/423) of these isolates. Other ARGs, such as *aac*(6′)‐*aph*(2”) and *blaZ*, were only found in fewer than 10 isolates (Figure S5). Given that most of the isolates were obtained from China (*n* = 208) and the United States (*n* = 139), we investigated the distribution of ARGs between these two regions. Our findings indicated that *C. perfringens* isolates from China carried a higher number of ARGs, which was primarily reflected in the significantly higher detection rates of *tetB*(P) and *erm*(Q) (Figure 5B).

Building on the additional gene clusters in CQ145 and their association with mobile genetic elements (MGEs) and phages, we selected another 55 human‐derived *C. perfringens* strains that shared over 99.99% sequence identity to identify MGE clusters. Notably, these strains were consistently sourced from the same country, suggesting potential regional transmission of *C. perfringens* strains among humans. Interestingly, we observed over 99.99% ANIs between CQ93 and MC066_D7 (Australia, Accession: GCF_951337105.1) and between CQ176 and 4928STDY7387889 (the UK, GCF_902166025.1), indicating possible trans‐regional transmission. Additionally, small genetic differences were commonly identified between the strains with >99.99% ANIs (Figure 5C), highlighting the prevalence of transfer of gene clusters in *C. perfringens* strains during the transmission.

### 2.6 Pan‐genomic analysis of *C. perfringens* from humans and animals

**Figure 6:**
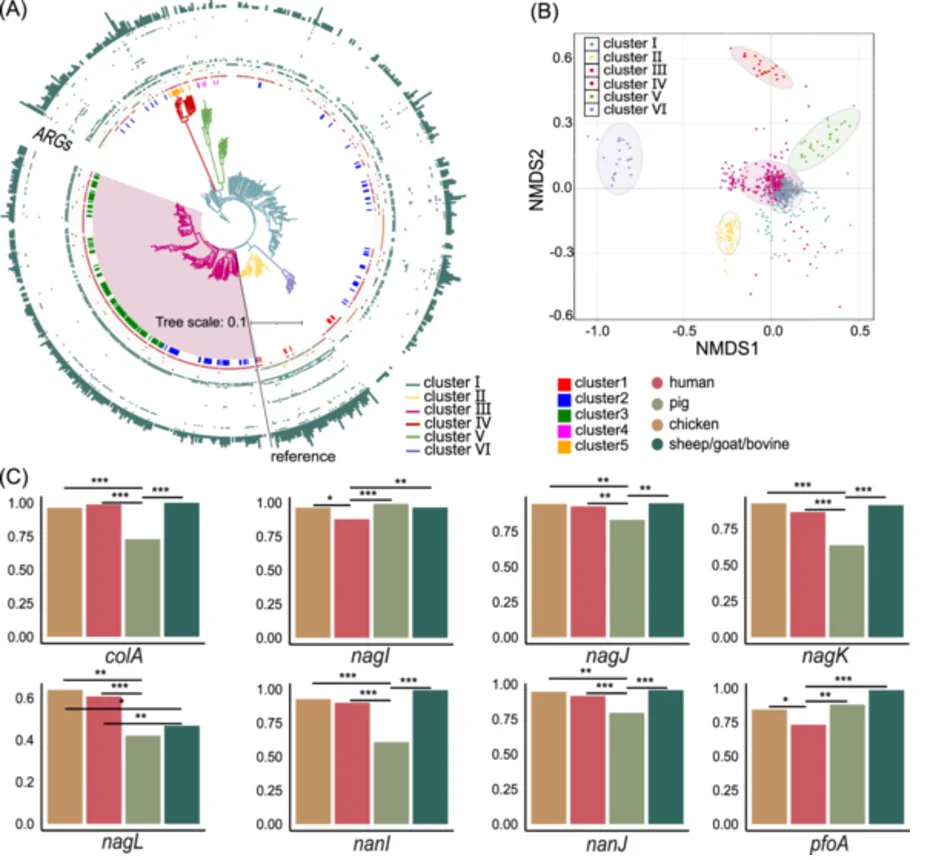
Population structure and pan‐genomic analysis of human‐derived and animal‐derived *Clostridium perfringens* strains worldwide. (A) Evolutionary relationship of *C. perfringens* from humans and animals. (B) Nonmetric multidimensional scaling (NMDS) analysis of the accessory gene profiles of strains from different clusters. (C) Detection rates of toxin genes *colA*, *nagI*, *nagJ*, *nagK*, *nagL*, *nanI*, *nanJ*, and *pfoA* in *C. perfringens* strains from different sources.

Through phylogenetic analysis of human‐derived isolates worldwide, we found that regional sources did not influence the evolutionary relationship of *C. perfringens* (Figure 5A). Therefore, we investigated the evolutionary relationship and accessory gene similarity of *C. perfringens* in a One Health context, encompassing isolates from humans (*n* = 423), pigs (*n* = 107), chickens (*n* = 108), and sheep/goat/bovine (*n* = 137) worldwide (Table S7). As expected, pigs emerged primary reservoir of ARGs (Figure S6). In the maximum‐likelihood tree based on 108,036 core‐genome SNPs, *C. perfringens* strains were divided into six evolutionary clusters, denoted Ⅰ to Ⅵ (Figure 6A). A high proportion of the human‐derived strains from China and other countries clustered in cluster Ⅲ. Nonmetric multidimensional scaling (NMDS) and cluster analyses of the accessory genes across different evolutionary clusters indicated that the strains within the same evolutionary cluster shared similar accessory gene profiles (Figures 6B and S7A).

Compared to the *C. perfringens* from China, one additional evolutionary cluster was identified. However, expansion of the regional sources did not change the basic structure of the maximum‐likelihood tree, and the China‐origin strains in clusters 1 to 5 were found to be correspondingly distributed in clusters Ⅰ to Ⅵ. This suggests that regional sources were not the primary factor determining the genomic characteristic of *C. perfringens*. Similar to the *C. perfringens* strains isolated in China, some strains from humans and animals worldwide were found to cluster together in cluster Ⅰ. However, the other *C. perfringens* strains of the same animal origin clustered together in the phylogenetic tree, and a high proportion of the strains from humans, pigs, and chickens were found to share similar accessory gene profiles, respectively (Figure S7B). Furthermore, higher ANIs were also identified among the strains from the same animal origin, especially pigs (Figure S8). These results collectively indicate a clear link between host species and the genomic characteristics of *C. perfringens*.

The virulence of *C. perfringens* is largely attributed to their protein toxins and enzymes. To gain preliminary insights into the genetic differences between the *C. perfringens* from different animals, we investigated the prevalence of chromosome‐borne toxin genes, excluding *plc*, which is present in all *C. perfringens* strains. Our results indicated no significant differences in the detection rates of *cloSI*, *nagH*, and *nanH* across animal hosts; however, the detection rates of other toxin genes varied by host species (Figure 6C). Specifically, a lower proportion of the human‐derived strains were positive for *pfoA* and *nagI*, while detection rates of *colA*, *nagK*, and *nanI* were significantly lower in pig‐derived strains. Additionally, *nagL* appeared to be an atypical virulence factor in *C. perfringens*, as it could only be detected in ~50% of the *C. perfringens* strains, in contrast to other toxin genes.

## 3 DISCUSSION

*C. perfringens* is a significant foodborne pathogen that poses a substantial threat to public health. Several intestinal illnesses, including AAD, have been linked to *C. perfringens* infections [27]. In this study, we investigated the genomic characteristics of human‐derived *C. perfringens* from China and performed phylogenetic and pan‐genomic analyses in conjunction with other available strains worldwide.

Our findings revealed that the primary ARGs among the *C. perfringens* isolates are *tetA*(P), *tetB*(P), *erm*(Q), and *lnu*(P), corresponding to resistance rates of 57.9% to erythromycin, 50.7% to clindamycin, and moderate resistance of 32.0% to tetracycline (Table S1). Previous studies have established the association between ARGs, particularly *erm*(Q), *tetA*(P), and *optrA*, and the pCW3‐like and pCPCP‐like plasmids in *C. perfringens* [9, 11, 28, 29]. However, in our analysis, ARGs were found in *C. perfringens* strains with various plasmid profiles, regardless of the presence of the pCW3‐like and pCPCP‐like plasmids. This suggests that ARGs are distributed across diverse genetic locations and may spread through multiple transmission routs. As a significant foodborne pathogen, the antibiotic resistance of *C. perfringens* poses a notable public health concern. Furthermore, ARGs in *C. perfringens* have the potential to transfer to other bacterial species, contributing to the broader dissemination of antibiotic resistance [9]. The prevalence of ARGs in *C. perfringens*, particularly *erm*(Q), which is frequently detected in human‐derived strains in China, underscores the need for active surveillance.

*C. perfringens* toxins CPE and BEC have been considered as the primary causing factors of food poisoning [18]. In this study, genes encoding *cpe‐* and *becA/B* were detected in four and one *C. perfringens* strains, respectively. The *cpe* is known to be located on both the chromosome and pCW3‐like plasmids [30]. Interestingly, our analysis revealed the co‐localization of *cpe* with △IS*1469*, IS*1151*, and *dcm* in isolates CQ147, CQ165, and CQ169, respectively. This observation supports the hypothesis that *cpe*‐containing plasmids in *C. perfringens* may originate from the integration of a *cpe*‐bearing genetic element near the *dcm* loci of *C. perfringens* plasmids [31]. In contrast, in isolate CQ145, *cpe* was located on a fusion plasmid along with △IS*1469* and IS*1151*, but not with *dcm*. This suggests that plasmid fusion contributed to the novel genetic background of *cpe* in *C. perfringens*.

The transmission of pathogenic microorganisms is a leading cause of foodborne disease outbreaks. Some *C. perfringens* isolates from this study, along with the human‐derived strains from other countries, shared > 99.99% ANI, suggesting potential regional/transregional transmission of *C. perfringens* in humans. Furthermore, we found that small genetic variances between these strains were common, indicating frequent gene transfers during the transmission of *C. perfringens* strains.

CPB2 is encoded by either *cpb2* gene or the atypical *cpb2* (*aty.cpb2*) gene. The atypical gene has 60%–80% DNA identity to *cpb2*, but shows 99% identity with other *aty.cpb2* genes. However, a significant number of *aty.cpb2* genes contain a frameshift mutation that disrupt their expression [32]. Previous studies have identified pigs as the main reservoir of *cpb2*, whereas the *aty.cpb2* gene is predominantly detected in *C. perfringens* isolates of non‐porcine sources [9, 32, 33], suggesting a potential host‐specific association with *C. perfringens*. However, no comparative study has been conducted to explore whether there is a correlation between *C. perfringens* and host species. In this study, we performed a genomic investigation of *C. perfringens* isolates from humans in China, followed by phylogenetic and pan‐genomic analyses, including other available *C. perfringens* strains from humans and animals worldwide. The results showed that while only certain strains from humans and animals clustered together in the evolution tree, a substantial proportion of strains from the same animal source clustered together in the phylogenetic tree and shared similar accessory gene profiles. These findings indicate a strong clonal association of *C. perfringens* strains within the same host species.

The presence of genetic markers in a population often reflects interactions between organisms and their external environments. In this study, we observed variations in certain toxin genes, such as *pfoA, nanI*, and *colA*. A recent study demonstrated that *pfoA* is largely deficient in a human‐derived hypovirulent lineage, contrasting with its presence in typical *pfoA*‐encoding virulent lineages [5]. Given the lower detection rate of *pfoA* in the isolates from humans, we speculated that: (i) *C. perfringens* strains have adapted to achieve symbiosis with specific host animals by selectively discarding specific toxin genes to reduce virulence; and (ii) variations in intestinal physiology and composition across animal species may facilitate colonization by strains with unique biological traits. Nevertheless, the decreased prevalence of *colA*, *nanI*, *nanJ*, and *nagI* in pigs and humans suggests these genes might play essential roles in the pathogenesis of *C. perfringens* in the hosts. Further research is warranted to elucidate the biological functions of these toxin genes.

## 4 CONCLUSION

In conclusion, this study comprehensively investigated the genomic characteristics of *C. perfringens* isolates from humans in China, as well as the population structure and pan‐genomic characteristics of *C. perfringens* worldwide. The findings offer novel insights into the transmission dynamics of *C. perfringens* strains, the localization of key virulence markers, and a relationship between *C. perfringens* and host species. Moreover, our results suggest that toxin genes may play a crucial role in determining the host selectivity of *C. perfringens*, highlighting the importance of further research into the molecular mechanisms underlying this pathogen's virulence.

## 5 MATERIAL AND METHODS

### 5.1 Collection of the *Clostridium perfringens* strains

A total of 220 *C. perfringens* strains were obtained from 426 healthy individuals and 273 intensive care unit (ICU) patients at the Second Affiliated Hospital of Zhejiang University College of Medicine, Hangzhou, China. Healthy adults who were considered generally healthy during their routine physical examinations were consented and included in the study. Exclusion criteria for this group included gastroenteritis, pregnancy, and subjects receiving antibiotic treatments in the preceding 30 days. Meanwhile, ICU patients who have been hospitalized for at least 7 days were included with informed consent provided by their guardians. The isolation and identification methods for the *C. perfringens* strains have been described in a previous study [34]. Each sample was streaked onto tryptose‐sulfite‐cycloserine agar (Land Bridge) and incubated anaerobically at 37°C for 20–24 h. Suspected colonies were subsequently purified on 5% defibrinated sheep blood agar and definitively identified as *C. perfringens* using matrix‐assisted laser desorption ionization‐time‐of‐flight mass spectrometry (Bruker Daltonik GmbH).

### 5.2 Genome sequencing

Genomic DNA of the *C. perfringens* isolates was extracted using a PureLink Genomic DNA mini kit (Invitrogen). Indexed DNA libraries were prepared using a TruSeq DNA PCR‐free sample preparation kit (Illumina, Inc.), 300‐bp paired‐end reads with a minimum of 150‐fold coverage for each isolate were obtained following sequencing using the Illumina HiSeq X Ten System. Raw reads were processed by trimming and assembling into contigs using SPAdes version 3.11.1 [35]. Genome quality was assessed using checkM v.1.0.12 [36] and only genomes with >95% completeness and <5% contamination were included in the subsequent genomic analysis.

### 5.3 Screening for genetic markers

Given the clinical association of antibiotic resistance, virulence, and plasmids of *C. perfringens*, we performed analyses to detect toxin genes, ARGs, pCW3‐like and pCP13‐like plasmids, and the novel pCPCPI53k‐r1_1‐like (abbreviated as pCPCP‐like) plasmids [9] in the isolates. Toxin genes and ARGs were screened using ABRicate against ResFinder (https://github.com/cadms/resfinder), VFDB (http://www.mgc.ac.cn/VFs/) databases (‐‐minid 90 ‐‐mincov 90). Tcp (the conjugative locus of pCW3‐like plasmids) and Pcp loci (the conjugative locus of pCP13‐like plasmids) were identified using ABRicate, utilizing a custom‐built genetic database. Tcp (http://www.ncbi.nlm.nih.gov/nuccore/NC_010937.1) and Pcp (http://www.ncbi.nlm.nih.gov/nuccore/AP003515.1) loci coding genes were used as blast database. The strains harboring more than three Tcp or Pcp locus coding genes were regarded as harboring the according plasmids. Pan‐genomic analysis was used as a secondary approach to determine the existence of pCW3‐like and pCP13‐like plasmids among the *C. perfringens* genomes. Plasmids pCW3 and pCP13 and the 195 sequenced *C. perfringens* isolates in this study were included in this analysis. pCPCP‐like plasmids were identified by pan‐genomic analysis using Roary v3.13.0 [37] and FriPan (https://github.com/drpowell/FriPan), the identified pCPCP‐like plasmids p21‐D‐5b (NZ_CP119189.1), pQHY‐2 (NZ_CP118266.1), p2C45 (NZ_JAAQTM010000004.1), and pCPCPI53k‐r1_1 (NZ_CP075935.1) were used as references. In all pan‐genomic analyses, the minimum sequence identity threshold for BLASTP was set at 95%.

### 5.4 Investigation of genetic environments of *cpe* and *becA/B*

The *cpe* and *becA/B* genes are crucial virulence factors linked to foodborne infections in humans [18, 38]. To deepen the understanding of these clinical important genetic markers, we investigated the genetic environments of *cpe* and *becA/B* among the *C. perfringens* isolates. Sequence annotations of the segments positive for *cpe* or *becA/B* were performed using online tool RAST (Rapid Annotation using Subsystem Technology, https://rast.nmpdr.org/), insertion sequences (ISs) were screened using ISfinder (https://isfinder.biotoul.fr/about.php) and DANMEL [39]. Sequence comparisons were performed using EasyFig. 2.2.2 [40] and BRIG 0.95 [41]. The genetic environment of *cpe* in isolate CQ145 was validated by PCR assays, and the PCR products were sequenced using Sanger sequencing.

### 5.5 Evolutionary analysis

To uncover the potential transmission of the *C. perfringens* strains and the population structure of *C. perfringens*, this study constructed maximum‐likelihood trees based on core‐genome SNPs. From the *C. perfringens* strains in China and worldwide. Snippy v4.6.0 (https://github.com/tseemann/snippy) was used to call core‐genome SNPs from the *C. perfringens* strains. Phylogenetic trees were constructed using IQ‐TREE 2 [42], with the parameter “‐m MF”. The resulting trees were grouped using rhierBAPS [43] and annotated using the online tool iTOL (https://itol.embl.de/).

### 5.6 Pan‐genomic analysis

Most of the primary toxin genes and ARGs belong to accessory genes, indicating the importance and necessity of pan‐genomic analysis. Genome annotation of the *C. perfringens* strains was performed using Prokka v1.14.5 [44]. Subsequently, Roary v3.13.0 and IPGA [45] were used to conduct the pan‐genomic analysis. Accessory genes present in 5% to 95% of the genomes were selected for comparison, allowing for the assessment of differences in accessory gene profiles across groups.

### 5.7 Statistical analysis

All statistical analyses were performed using R version 4.3.2. NMDS analysis of accessory gene was performed using vegan (https://github.com/vegandevs/vegan) and plotted using ggplot2 (https://github.com/tidyverse/ggplot2). Differences in the total number of ARGs and virulence genes among the *C. perfringens* strains from various groups were assessed using the Wilcoxon test. The Pearson chi‐square (*χ* ^2^) test was used to test whether differences in frequencies of individual genes encoding virulence and drug resistance phenotypes were significant. *p < 0.05, **p < 0.01, ***p < 0.001.

## AUTHOR CONTRIBUTIONS

**Hongning Wang**: Concept; design; and supervision. **Rong Zhang**: Concept; design; and supervision. **Zhe Yin**: Concept; design; and supervision. **Shaolin Wang**: Methodology and data analysis. **Yang Wang**: Methodology and data analysis. **Ruichao Li**: Methodology and data analysis. **Li Bai**: Methodology and data analysis. **Chengtao Sun**: Methodology; data analysis; investigation; and data curation. **Bo Fu**: Methodology and data analysis. **Yanyan Zhu**: Investigation and data curation. **Zelin Yan**: Investigation and data curation. **Ke Wu**: Writing–initial manuscript. **Juan Wang**: Writing–initial manuscript; review and editing. **Séamus Fanning**: Review and editing. **Edward M. Fox**: Review and editing. **Yizhi Tang**: Review and editing. All authors have read the final manuscript and approved it for publication.

## CONFLICT OF INTEREST STATEMENT

The authors declare no conflict of interest.

## ETHICS STATEMENT

No animals or humans were involved in this study.

## Supporting information


**Figure S1.** Polymerase Chain Reaction (PCR) identification of the junction on the fusion plasmid pcq145‐tcppcp. **Figure S2.** Sequence analysis and formation of the fusion plasmid pcq145‐tcppcp. **Figure S3**. Sequence comparison between plasmid pCP‐TS1 and the *becA/B*‐positive contig of *C. perfringens* strain CQ92. **Figure S4.** Genomic comparison of human‐derived *C. perfringens* strains from different countries. **Figure S5.** Antibiotic resistance genes (ARGs) of the human‐derived strains. **Figure S6.** Comparison of the total number of ARGs in *C. perfringens* strains from humans and animals. **Figure S7.** Comparison of accessory gene profiles of *C. perfringens* strains across different phylogenetic clusters and sources. **Figure S8.** Average nucleotide identity (ANI) of *C. perfringens* strains from different sources.

**Table S1.** Source and antibiotic resistance of the 195 sequenced *Clostridium perfringens* isolates in this study. **Table S2.** Identification of Pcp and Tcp loci coding genes. **Table S3.** Pan‐genomic analysis of plasmids pCW3 and pCP13 and the 195 *C. perfringens* isolates in this study. **Table S4.** Top ten blast results of segment 1. **Table S5.** Source, toxin genes, and antibiotic resistance genes (ARGs) of *C. perfringens* strains in China. **Table S6.** Source, toxin genes, and ARGs of *C. perfringens* strains from humans. **Table S7.** Source, toxin genes, and ARGs of *C. perfringens* strains from pigs, chicken, and sheep/goat/bovine worldwide.

## Data Availability

The data that support the findings of this study are available from the corresponding author upon reasonable request. All the sequencing data have been deposited in NCBI under BioProject accession number PRJNA983292. The data and scripts were saved at https://github.com/s-ckk/data-and-scripts-for-IMO-2024-0069. Supplementary materials (figures, tables, graphical abstract, slides, videos, Chinese translated version and update materials) may be found in the online DOI or iMetaOmics, http://www.imeta.science/imetaomics/.

## References

[1] Li, Jihong , Vicki Adams , Trudi L. Bannam , Kazuaki Miyamoto , Jorge P. Garcia , Francisco A. Uzal , Julian I. Rood , and Bruce A. McClane . 2013. “Toxin Plasmids of *Clostridium perfringens* .” Microbiology and Molecular Biology Reviews 77: 208–233. 10.1128/MMBR.00062-12 PMC3668675 23699255

[2] Kiu, Raymond , and Lindsay J. Hall . 2018. “An Update on the Human and Animal Enteric Pathogen *Clostridium perfringens* .” Emerging Microbes & Infections 7: 1–15. 10.1038/s41426-018-0144-8 PMC6079034 30082713

[3] Rood, Julian I. , Vicki Adams , Jake Lacey , Dena Lyras , Bruce A. McClane , Stephen B. Melville , Robert J. Moore , et al. 2018. “Expansion of the *Clostridium perfringens* Toxin‐Based Typing Scheme.” Anaerobe 53: 5–10. 10.1016/j.anaerobe.2018.04.011 PMC6195859 29866424

[4] Hamza, Dalia , Sohad M. Dorgham , Mahmoud Elhariri , Rehab Elhelw , and Elshaimaa Ismael . 2018. “New Insight of Apparently Healthy Animals as a Potential Reservoir for *Clostridium perfringens*: A Public Health Implication.” Journal of Veterinary Research 62: 457–462. 10.2478/jvetres-2018-0073 PMC6364162 30729202

[5] Kiu, Raymond , Alexander G. Shaw , Kathleen Sim , Antia Acuna‐Gonzalez , Christopher A. Price , Harley Bedwell , Sally A. Dreger , et al. 2023. “Particular Genomic and Virulence Traits Associated with Preterm Infant‐Derived Toxigenic *Clostridium perfringens* Strains.” Nature Microbiology 8: 1160–1175. 10.1038/s41564-023-01385-z PMC10234813 37231089

[6] Kiu, Raymond , Shabhonam Caim , Anais Painset , Derek Pickard , Craig Swift , Gordon Dougan , Alison E. Mather , Corinne Amar , and Lindsay J. Hall . 2019. “Phylogenomic Analysis of Gastroenteritis‐Associated *Clostridium perfringens* in England and Wales over a 7‐year Period Indicates Distribution of Clonal Toxigenic Strains in Multiple Outbreaks and Extensive Involvement of Enterotoxin‐Encoding (CPE) Plasmids.” Microb Genom 5: e000297. 10.1099/mgen.0.000297 PMC6861862 31553300

[7] Scallan, Elaine , Robert M. Hoekstra , Frederick J. Angulo , Robert V. Tauxe , Marc‐Alain Widdowson , Sharon L. Roy , Jeffery L. Jones , and Patricia M. Griffin . 2011. “Foodborne Illness Acquired in the United States‐‐Major Pathogens.” Emerging Infectious Diseases 17: 7–15. 10.3201/eid1701.p11101 PMC3375761 21192848

[8] Mellou, Kassiani , Maria Kyritsi , Anthi Chrysostomou , Theologia Sideroglou , Theano Georgakopoulou , and Christos Hadjichristodoulou . 2019. “ *Clostridium perfringens* foodborne outbreak during an athletic event in Northern Greece, June 2019.” International Journal of Environmental Research and Public Health 16: 3967. 10.3390/ijerph16203967 PMC6843328 31627449

[9] Wu, Ke , Zhe Li , Mingjin Fang , Yuan Yuan , Edward M. Fox , Yingqiu Liu , Ruichao Li , et al. 2023. “Genome Characteristics of the *optrA*‐positive *Clostridium perfringens* Strain QHY‐2 Carrying a Novel Plasmid Type.” mSystems 8: e0053523. 10.1128/msystems.00535-23 PMC10469678 37458450

[10] Gulliver, Emily L. , Vicki Adams , Vanessa Rossetto Marcelino , Jodee Gould , Emily L. Rutten , David R. Powell , Remy B. Young , et al 2023. “Extensive Genome Analysis Identifies Novel Plasmid Families in *Clostridium perfringens* .” Microbial Genomics 9: mgen000995. 10.1099/mgen.0.000995 PMC10210947 37079454

[11] Revitt‐Mills, Sarah A. , Thomas D. Watts , Dena Lyras , Vicki Adams , and Julian I. Rood . 2021. “The Ever‐Expanding tcp Conjugation Locus of pCW3 from *Clostridium perfringens* .” Plasmid 113: 102516. 10.1016/j.plasmid.2020.102516 32526229

[12] Yanagimoto, Keita , and Eiji Haramoto . 2021. “Isolation of Alpha‐Toxin‐Deficient *Clostridium perfringens* Type F from Sewage Influents and Effluents.” Microbiology Spectrum 9: e0021421. 10.1128/Spectrum.00214-21 PMC8552768 34259541

[13] Adams, Vicki , Xiaoyan Han , Dena Lyras , and Julian I. Rood . 2018. “Antibiotic Resistance Plasmids and Mobile Genetic Elements of *Clostridium perfringens* .” Plasmid 99: 32–39. 10.1016/j.plasmid.2018.07.002 30055188

[14] Watts, Thomas D. , Daouda A. K. Traore , Sarah C. Atkinson , Carmen Lao , Natalie Caltabiano , Julian I. Rood , and Vicki Adams . 2022. “The Specificity of ParR Binding Determines the Incompatibility of Conjugative Plasmids in *Clostridium perfringens* .” mBio 13: e0135622. 10.1128/mbio.01356-22 PMC9426499 35726914

[15] Li, Jihong , and Bruce A. McClane . 2021. “NanH is Produced by Sporulating Cultures of *Clostridium perfringens* Type F Food Poisoning Strains and Enhances the Cytotoxicity of *C. perfringens* Enterotoxin.” mSphere 6: e00176–21. 10.1128/mSphere.00176-21 PMC8092135 33910991

[16] Mehdizadeh Gohari, Iman , Jihong Li , Mauricio A. Navarro , Fábio S Mendonça , Francisco A. Uzal , and Bruce A. McClane . 2023. “Identification of Orphan Histidine Kinases That Impact Sporulation and Enterotoxin Production by *Clostridium perfringens* Type F Strain SM101 in a Pathophysiologically‐Relevant *ex vivo* Mouse Intestinal Contents Model.” PLoS Pathogens 19: e1011429. 10.1371/journal.ppat.1011429 PMC10263361 37262083

[17] Ueda, Kengo , Kazuki Kawahara , Narumi Kimoto , Yusuke Yamaguchi , Kazuhiro Yamada , Hiroya Oki , Takuya Yoshida , et al. 2022. “Analysis of the Complete Genome Sequences of *Clostridium perfringens* Strains Harbouring the Binary Enterotoxin BEC Gene and Comparative Genomics of pCP13‐like Family Plasmids.” BMC Genomics 23: 226. 10.1186/s12864-022-08453-4 PMC8941779 35321661

[18] Yonogi, Shinya , Shigeaki Matsuda , Takao Kawai , Tomoko Yoda , Tetsuya Harada , Yuko Kumeda , Kazuyoshi Gotoh , et al. 2014. “BEC, a Novel Enterotoxin of *Clostridium perfringens* Found in Human Clinical Isolates from Acute Gastroenteritis Outbreaks.” Infection and Immunity 82: 2390–2399. 10.1128/IAI.01759-14 PMC4019177 24664508

[19] Fontenele, Rafaela S. , Simona Kraberger , James Hadfield , Erin M. Driver , Devin Bowes , LaRinda A. Holland , Temitope O. C. Faleye , et al. 2021. “High‐Throughput Sequencing of SARS‐CoV‐2 in Wastewater Provides Insights into Circulating Variants.” Water Research 205: 117710. 10.1016/j.watres.2021.117710 PMC8464352 34607084

[20] Widström, Julia , Maria E. Andersson , Johan Westin , Martina Wahllöf , Magnus Lindh , and Gustaf E. Rydell . 2023. “Complex Norovirus Transmission Dynamics at Hospital Wards Revealed by Deep Sequencing.” Journal of Clinical Microbiology 61: e0060823. 10.1128/jcm.00608-23 PMC10662361 37889018

[21] Wu, Ke , Hang Feng , Jiangang Ma , Bin Wang , Jie Feng , Hui Zhang , Yanfen Jiang , et al. 2022. “Prevalence, Toxin‐Typing and Antimicrobial Susceptibility of *Clostridium perfringens* in Sheep with Different Feeding Modes from Gansu and Qinghai Provinces, China.” Anaerobe 73: 102516. 10.1016/j.anaerobe.2022.102516 35026419

[22] Li, Jiyun , Yuqing Zhou , Dawei Yang , Shan Zhang , Zhiliang Sun , Yang Wang , Shaolin Wang , and Congming Wu . 2020. “Prevalence and Antimicrobial Susceptibility of *Clostridium perfringens* in Chickens and Pigs from Beijing and Shanxi, China.” Veterinary Microbiology 252: 108932. 10.1016/j.vetmic.2020.108932 33316633

[23] Murphy, C. G. , and M. H. Malamy . 1995. “Requirements for Strand‐ and Site‐Specific Cleavage Within the *Orit* Region of Tn*4399*, a Mobilizing Transposon from *Bacteroides fragilis* .” Journal of Bacteriology 177: 3158–3165. 10.1128/jb.177.11.3158-3165.1995 PMC177006 7768814

[24] Lyras, Dena , Vicki Adams , Isabelle Lucet , and Julian I. Rood . 2004. “The Large Resolvase TnpX Is the Only Transposon‐Encoded Protein Required for Transposition of the Tn*4451/3* Family of Integrative Mobilizable Elements.” Molecular Microbiology 51: 1787–1800. 10.1111/j.1365-2958.2003.03950.x 15009902

[25] Wang, Hongmei , Margaret C. M. Smith , and Peter Mullany . 2006. “The Conjugative Transposon Tn*5397* Has a Strong Preference for Integration into Its *Clostridium difficile* Target Site.” Journal of Bacteriology 188: 4871–4878. 10.1128/JB.00210-06 PMC1483006 16788196

[26] Cao, Huiluo , Salim Bougouffa , Tae‐Jin Park , Andes Lau , Man‐Ki Tong , Kin‐Hung Chow , and Pak‐Leung Ho . 2022. “Sharing of Antimicrobial Resistance Genes between Humans and Food Animals.” mSystems 7: e0077522. 10.1128/msystems.00775-22 PMC9765467 36218363

[27] Talukdar, Prabhat K. , Pathima Udompijitkul , Ashfaque Hossain , and Mahfuzur R. Sarker . 2017. “Inactivation Strategies for *Clostridium perfringens* Spores and Vegetative Cells.” Applied and Environmental Microbiology 83: e02731–16. 10.1128/AEM.02731-16 PMC5165105 27795314

[28] Wu, Ke , Juan Wang , Hang Feng , Ruichao Li , Xinglong Wang , and Zengqi Yang . 2022. “Complete Genome Sequence and Characterization of *Clostridium perfringens* Type D Carrying *Optra*‐plasmid and Tn*6218*‐like Transposon.” Journal of Antimicrobial Chemotherapy 78: 311–313. 10.1093/jac/dkac393 36411256

[29] Santos, Rui Andre Nunes Dos , Jiryes Abdel‐Nour , Cathy McAuley , Sean C. Moore , Narelle Fegan , and Edward M. Fox . 2022. “ *Clostridium perfringens* Associated With Dairy Farm Systems Show Diverse Genotypes.” International Journal of Food Microbiology 382: 109933. 10.1016/j.ijfoodmicro.2022.109933 36166891

[30] Freedman, John , Archana Shrestha , and Bruce McClane . 2016. “ *Clostridium perfringens* Enterotoxin: Action, Genetics, and Translational Applications.” Toxins 8: 73. 10.3390/toxins8030073 PMC4810218 26999202

[31] Miyamoto, Kazuaki , Ganes Chakrabarti , Yosiharu Morino , and Bruce A. McClane . 2002. “Organization of the Plasmid *Cpe* Locus in *Clostridium perfringens* Type A Isolates.” Infection and Immunity 70: 4261–4272. 10.1128/IAI.70.8.4261-4272.2002 PMC128129 12117935

[32] Jost, B Helen , Stephen J. Billington , Hien T. Trinh , Dawn M. Bueschel , and J Glenn Songer . 2005. “Atypical *cpb2* Genes, Encoding Beta2‐toxin in *Clostridium perfringens* Isolates of Nonporcine Origin.” Infection and Immunity 73: 652–656. 10.1128/IAI.73.1.652-656.2005 PMC538998 15618211

[33] Chan, Gloria , Abdolvahab Farzan , Glenn Soltes , Vivian M. Nicholson , Yanlong Pei , Robert Friendship , and John F. Prescott . 2012. “The Epidemiology of *Clostridium perfringens* Type A on Ontario Swine Farms, With Special Reference to *cpb2*‐positive Isolates.” BMC Veterinary Research 8: 156. 10.1186/1746-6148-8-156 PMC3503845 22947389

[34] Yan, Zelin , Bo Fu , Yanyan Zhu , Yanyan Zhang , Yuchen Wu , Panfeng Xiong , Hongwei Zhou , et al. 2024. “High Intestinal Carriage of *Clostridium perfringens* in Healthy Individuals and ICU Patients in Hangzhou, China.” Microbiology Spectrum 12: e0338523. 10.1128/spectrum.03385-23 PMC11218483 38771047

[35] Bankevich, Anton , Sergey Nurk , Dmitry Antipov , Alexey A. Gurevich , Mikhail Dvorkin , Alexander S. Kulikov , Valery M. Lesin , et al. 2012. “SPAdes: a New Genome Assembly Algorithm and its Applications to Single‐Cell Sequencing.” Journal of Computational Biology 19: 455–477. 10.1089/cmb.2012.0021 PMC3342519 22506599

[36] Parks, Donovan H. , Michael Imelfort , Connor T. Skennerton , Philip Hugenholtz , and Gene W. Tyson . 2015. “CheckM: Assessing the Quality of Microbial Genomes Recovered from Isolates, Single Cells, and Metagenomes.” Genome Research 25: 1043–1055. 10.1101/gr.186072.114 PMC4484387 25977477

[37] Page, Andrew J. , Carla A. Cummins , Martin Hunt , Vanessa K. Wong , Sandra Reuter , Matthew T. G. Holden , Maria Fookes , et al. 2015. “Roary: Rapid Large‐Scale Prokaryote Pan Genome Analysis.” Bioinformatics 31: 3691–3693. 10.1093/bioinformatics/btv421 PMC4817141 26198102

[38] Li, Jihong , Arhat Pradhan , and Bruce A. McClane . 2023. “NanJ Is the Major Sialidase for *Clostridium perfringens* Type F Food Poisoning Strain 01E809.” Infection and Immunity 91: e0005323. 10.1128/iai.00053-23 PMC10269042 37212696

[39] Wang, Peng , Xiaoyuan Jiang , Kai Mu , Ying Jing , Zhe Yin , Yujun Cui , Cuidan Li , et al. 2022. “DANMEL: A Manually Curated Reference Database for Analyzing Mobile Genetic Elements Associated with Bacterial Drug Resistance.” mLife 1: 460–464. 10.1002/mlf2.12046 PMC10989931 38818485

[40] Sullivan, Mitchell J. , Nicola K. Petty , and Scott A. Beatson . 2011. “Easyfig: a Genome Comparison Visualizer.” Bioinformatics 27: 1009–1010. 10.1093/bioinformatics/btr039 PMC3065679 21278367

[41] Alikhan, Nabil‐Fareed , Nicola K. Petty , Nouri L. Ben Zakour , and Scott A. Beatson . 2011. “BLAST Ring Image Generator (BRIG): Simple Prokaryote Genome Comparisons.” BMC Genomics 12: 402. 10.1186/1471-2164-12-402 PMC3163573 21824423

[42] Minh, Bui Quang , Heiko A. Schmidt , Olga Chernomor , Dominik Schrempf , Michael D. Woodhams , Arndt von Haeseler , and Robert Lanfear . 2020. “IQ‐TREE 2: New Models and Efficient Methods for Phylogenetic Inference in the Genomic Era.” Molecular Biology and Evolution 37: 1530–1534. 10.1093/molbev/msaa015 PMC7182206 32011700

[43] Cheng, Lu , Thomas R. Connor , Jukka Siren , David M. Aanensen , and Jukka Corander . 2013. “Hierarchical and Spatially Explicit Clustering of DNA Sequences with BAPS Software.” Molecular Biology and Evolution 30: 1224–1228. 10.1093/molbev/mst028 PMC3670731 23408797

[44] Seemann, Torsten . 2014. “Prokka: Rapid Prokaryotic Genome Annotation.” Bioinformatics 30: 2068–2069. 10.1093/bioinformatics/btu153 24642063

[45] Liu, Dongmei , Yifei Zhang , Guomei Fan , Dingzhong Sun , Xingjiao Zhang , Zhengfei Yu , Jinfeng Wang , et al. 2022. “IPGA: A Handy Integrated Prokaryotes Genome and Pan‐Genome Analysis Web Service.” iMeta 1: e55. 10.1002/imt2.55 PMC10989949 38867900

