# Supplementary material for: Genomic adaptation of Clostridium perfringens to human intestine
Source: IMetaOmics. 2024 Oct 25;1(2):e38. doi: 10.1002/imo2.38 (PMC12806229; doi:10.1002/imo2.38)
Supplement: Supplementary file 1 — Figure S1. Polymerase Chain Reaction (PCR) identification of the junction on the fusion plasmid pcq145‐tcppcp. Figure S2. Sequence analysis and formation of the fusion plasmid pcq145‐tcppcp. Figure S3. Sequence comparison between plasmid pCP‐TS1 and the becA/B‐positive contig of C. perfringens strain CQ92. Figure S4. Genomic comparison of human‐derived C. perfringens strains from different countries. Figure S5. Antibiotic resistance genes (ARGs) of the human‐derived strains. Figure S6. Comparison of the total number of ARGs in C. perfringens strains from humans and animals. Figure S7. Comparison of accessory gene profiles of C. perfringens strains across different phylogenetic clusters and sources. Figure S8. Average nucleotide identity (ANI) of C. perfringens strains from different sources. [file IMO2-1-e38-s001.docx]

**Supporting information to**

**Genomic adaptation of *Clostridium perfringens* to human intestine**

**Running title**: Host adaptation of *Clostridium perfringens*

Ke Wu^1#^, Juan Wang^2,3#^, Zelin Yan^4^, Yanyan Zhu^4^, Shaolin Wang^5^, Bo Fu^5^, Chengtao Sun^5^, Ruichao Li^6^, Edward M Fox^7^, Séamus Fanning^3,8^, Li Bai^3^, Yang Wang^5^, Yizhi Tang^1^, Zhe Yin^9*^, Rong Zhang^4*^, Hongning Wang^1*^

^1^Key Laboratory of Bio-Resource and Eco-Environment of Ministry of Education, College of Life Sciences, Sichuan University, Chengdu 610000, China

^2^Department of Preventive Veterinary Medicine, College of Veterinary Medicine, Northwest A&F University, Yangling 712100, China

^3^Research Unit of Food Safety, Chinese Academy of Medical Sciences (No. 2019RU014); NHC Key Lab of Food Safety Risk Assessment, China National Center for Food Safety Risk Assessment (CFSA), Beijing 100021, China

^4^Department of Clinical Laboratory, Second Affiliated Hospital of Zhejiang University, School of Medicine, Hangzhou 310000, China

^5^National Key Laboratory of Veterinary Public Health and Safety, College of Veterinary Medicine, China Agricultural University, Beijing 100193, China

^6^College of Veterinary Medicine, Yangzhou University, Yangzhou 225009, Jiangsu, China

^7^Department of Applied Sciences, Northumbria University, Newcastle upon Tyne NE1 8ST, UK

^8^UCD-Centre for Food Safety, School of Public Health, Physiotherapy and Sports Science, University College Dublin, Belfield, Dublin D04 N2E5, Ireland

^9^State Key Laboratory of Pathogen and Biosecurity, Academy of Military Medical Science, Beijing 100071, China

^#^These authors contributed equally: Ke Wu, Juan Wang

*Correspondence: [whongning@163.com](mailto:whongning@163.com) (Wang Hongning); [zhang-rong@zju.edu.cn](mailto:zhang-rong@zju.edu.cn) (Zhang Rong); [jerry9yin@163.com](mailto:jerry9yin@163.com) (Yin Zhe)

**
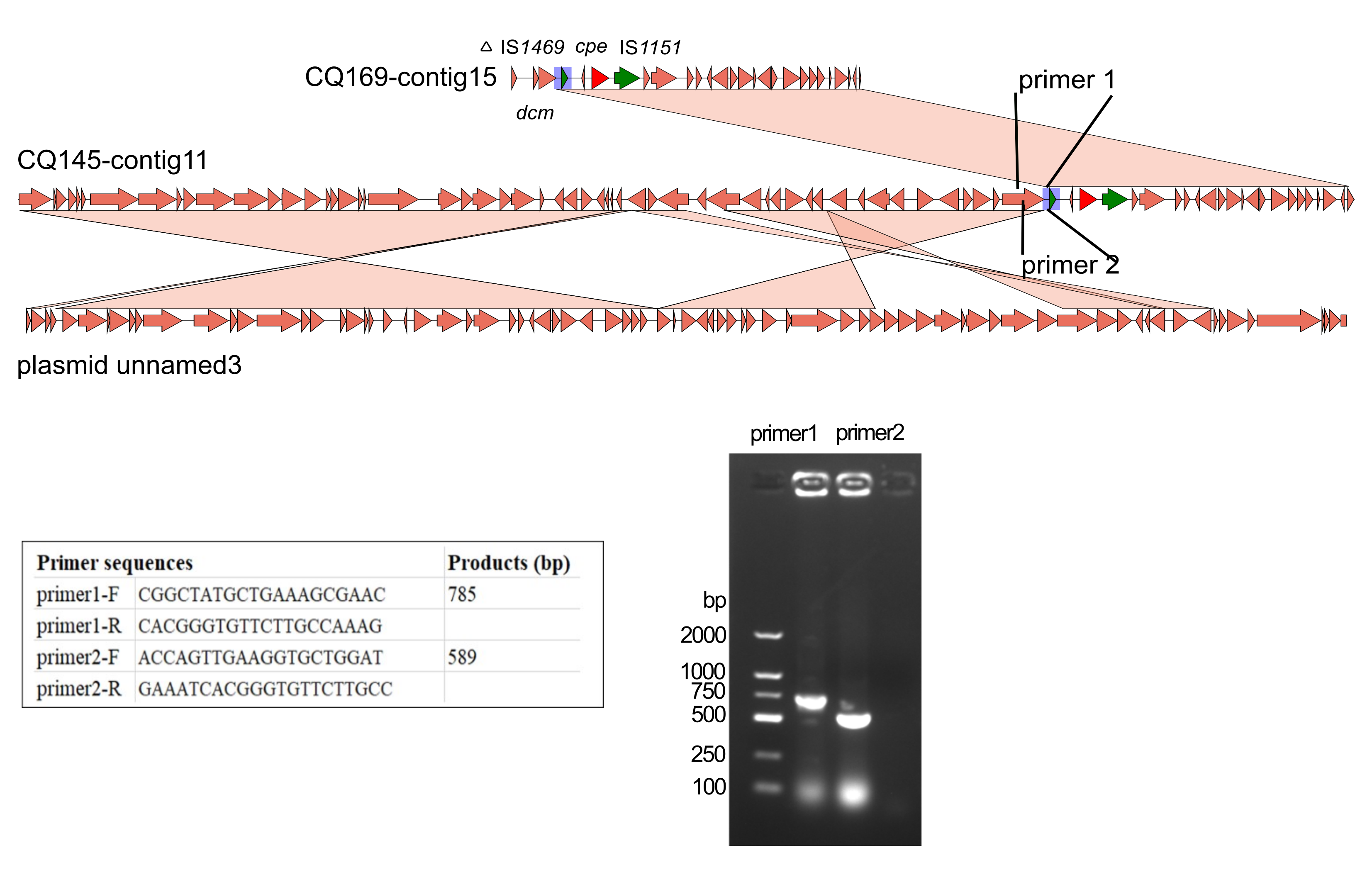
** **Figure S1** Polymerase Chain Reaction (PCR) identification of the junction on the fusion plasmid pcq145-tcppcp. Arrows indicate the transcription directions of the genes, and shaded regions denote shared sequences.


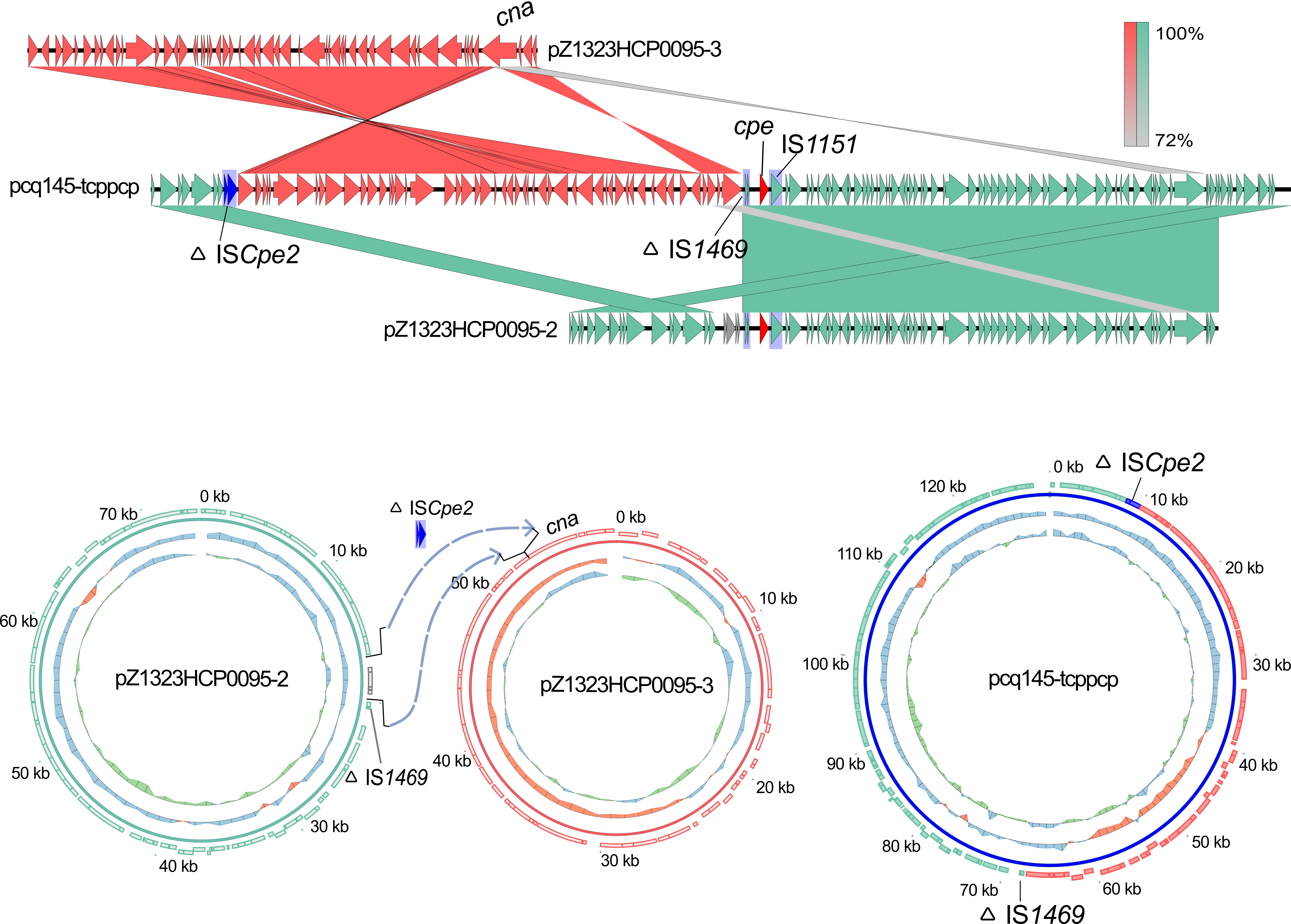


**Figure S2** Sequence analysis and formation of the fusion plasmid pcq145-tcppcp. Linear sequences are employed to compare the plasmids pZ1323HCP0095-2, pZ1323HCP0095-3, and pcq145-tcppcp. Arrows indicate the transcription directions of the genes, while shaded regions highlight shared sequences. Circular sequences are used to illustrate the formation diagram of pcq145-tcppcp.

**
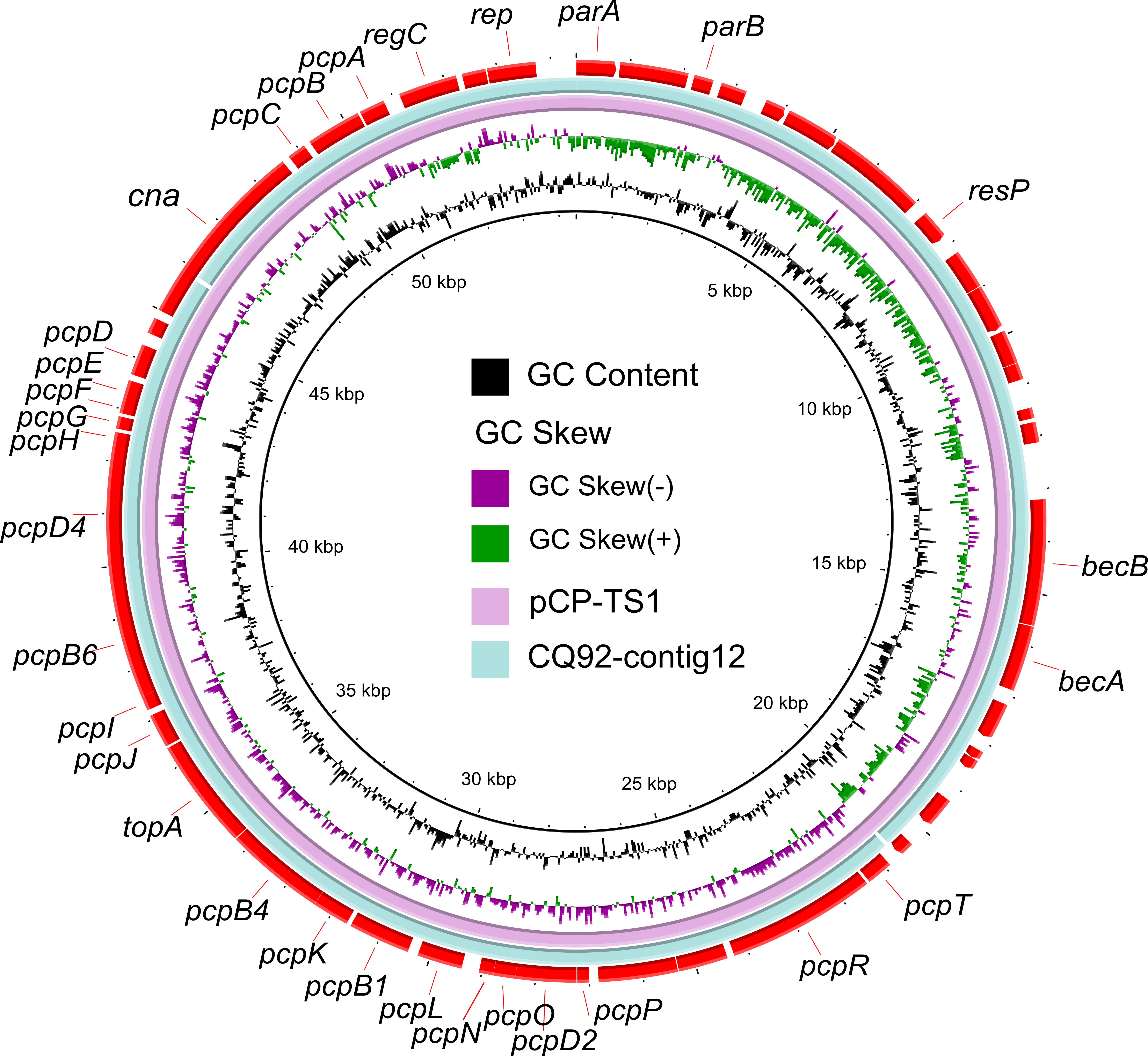
**

**Figure S3** Sequence comparison between plasmid pCP-TS1 and the *becA/B*-positive contig of *C. perfringens* strain CQ92. The annotation of pCP-TS1 is shown in the outermost ring, the secondary outer ring represents the *becA/B*-positive contig, and the third outer ring represents plasmid pCP-TS1.

**
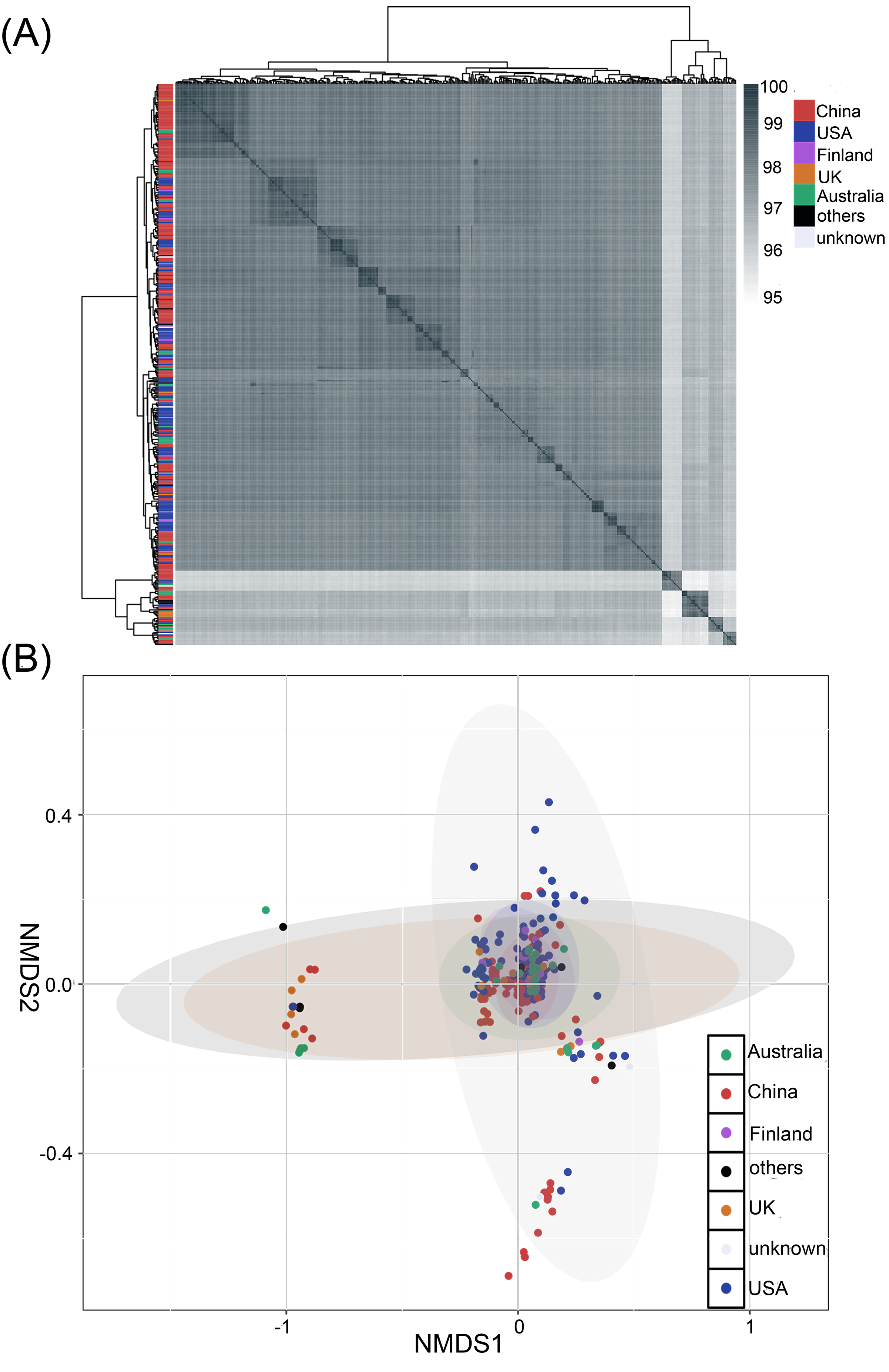
**

**Figure S4** **Genomic comparison of human-derived *C. perfringens* strains from different countries.** (A) Average nucleotide identity (ANI) among 423 *C. perfringens* strains from humans worldwide, with strains from different countries represented by distinct colors. (B) Non-metric multidimensional scaling (NMDS) analysis of the accessory gene profiles (present in 5%-95% of the strains) for *C. perfringens* strains from different countries.

*
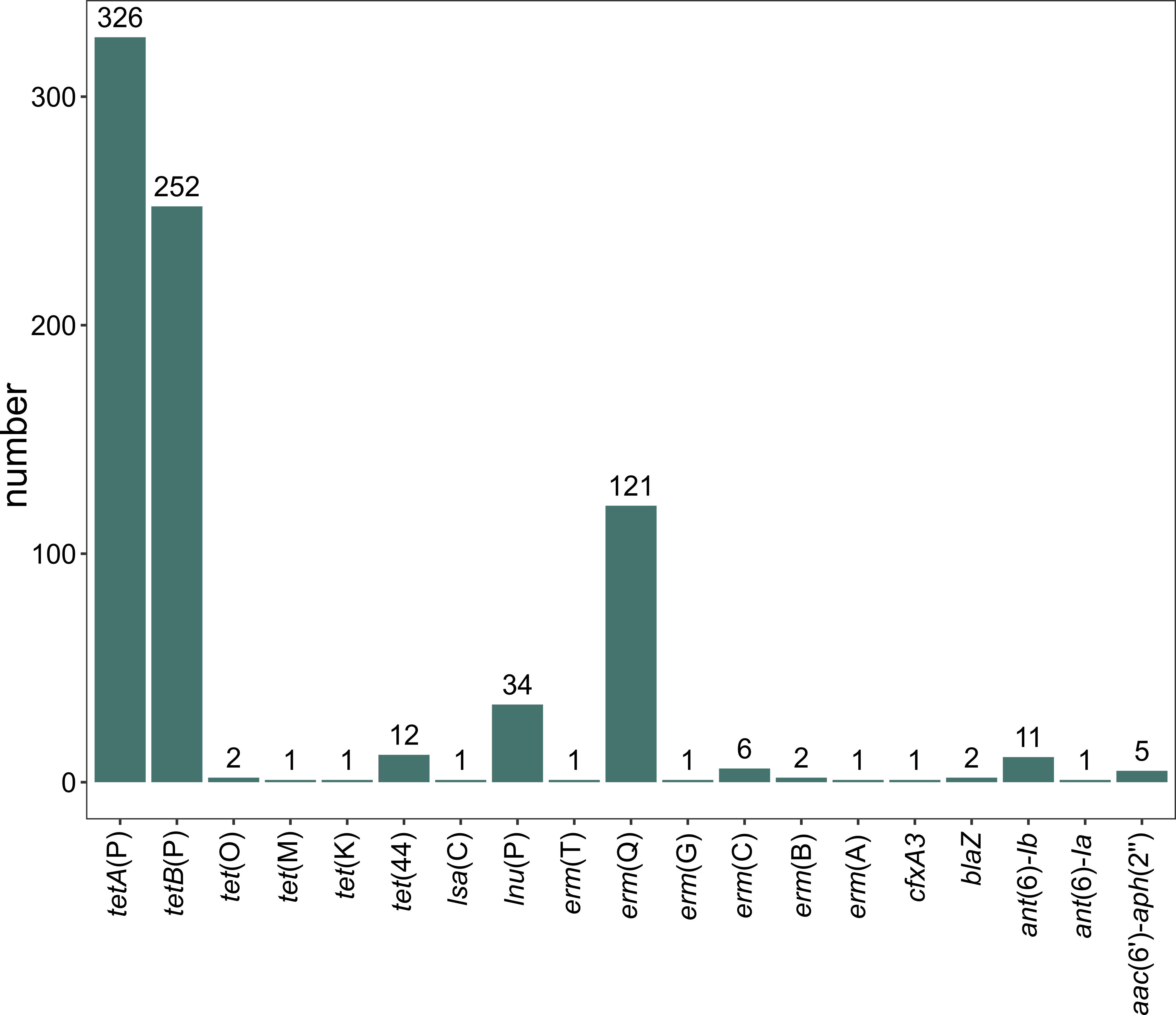
*

**Figure S5** ARGs of the human-derived strains. The X-axis represents the ARGs, while the Y-axis indicates the number of each gene.

*
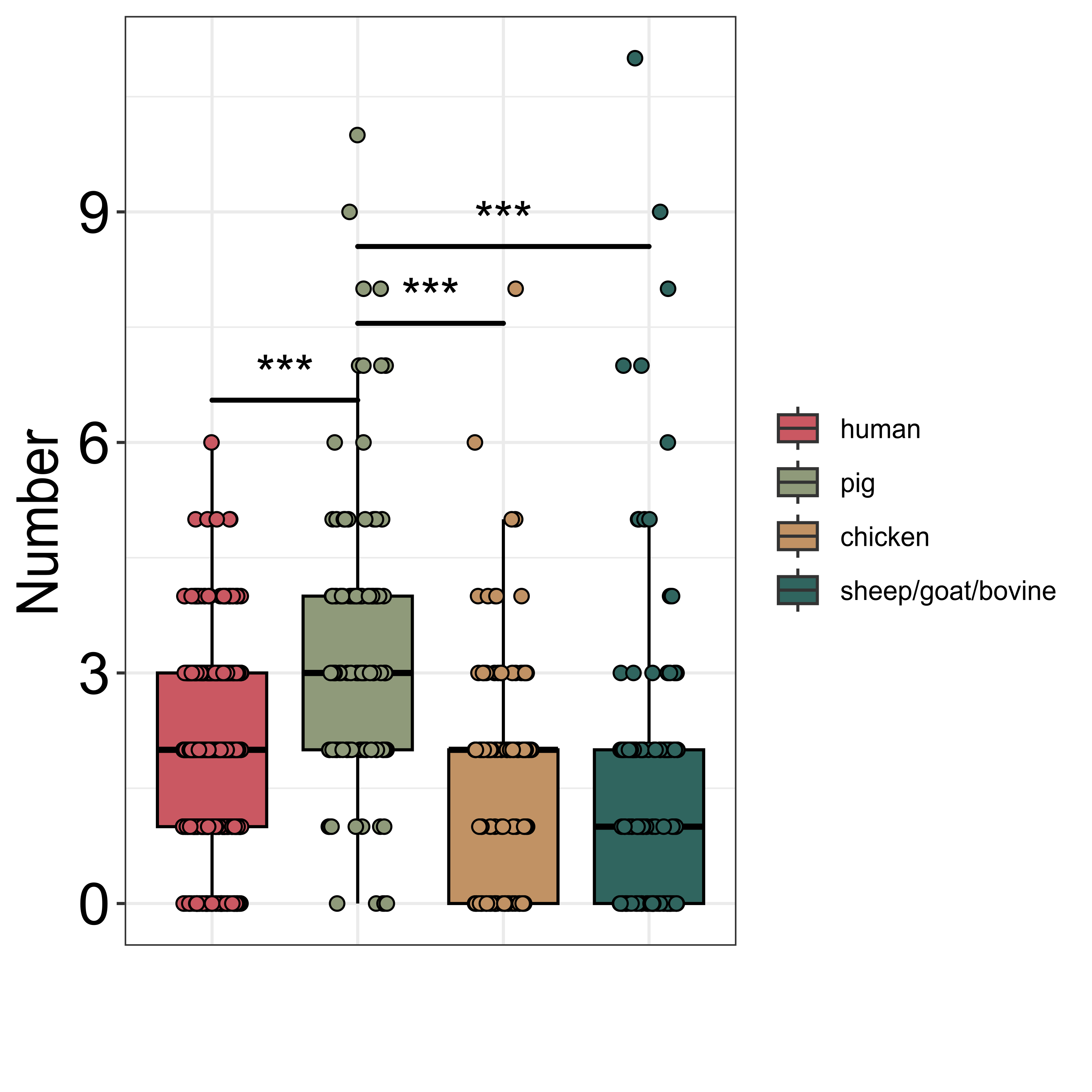
*

**Figure S6** Comparison of the total number of ARGs in *C. perfringens* strains from humans and animals. *C. perfringens* strains from different sources are represented with different colors. ***: *p* < 0.001.

**
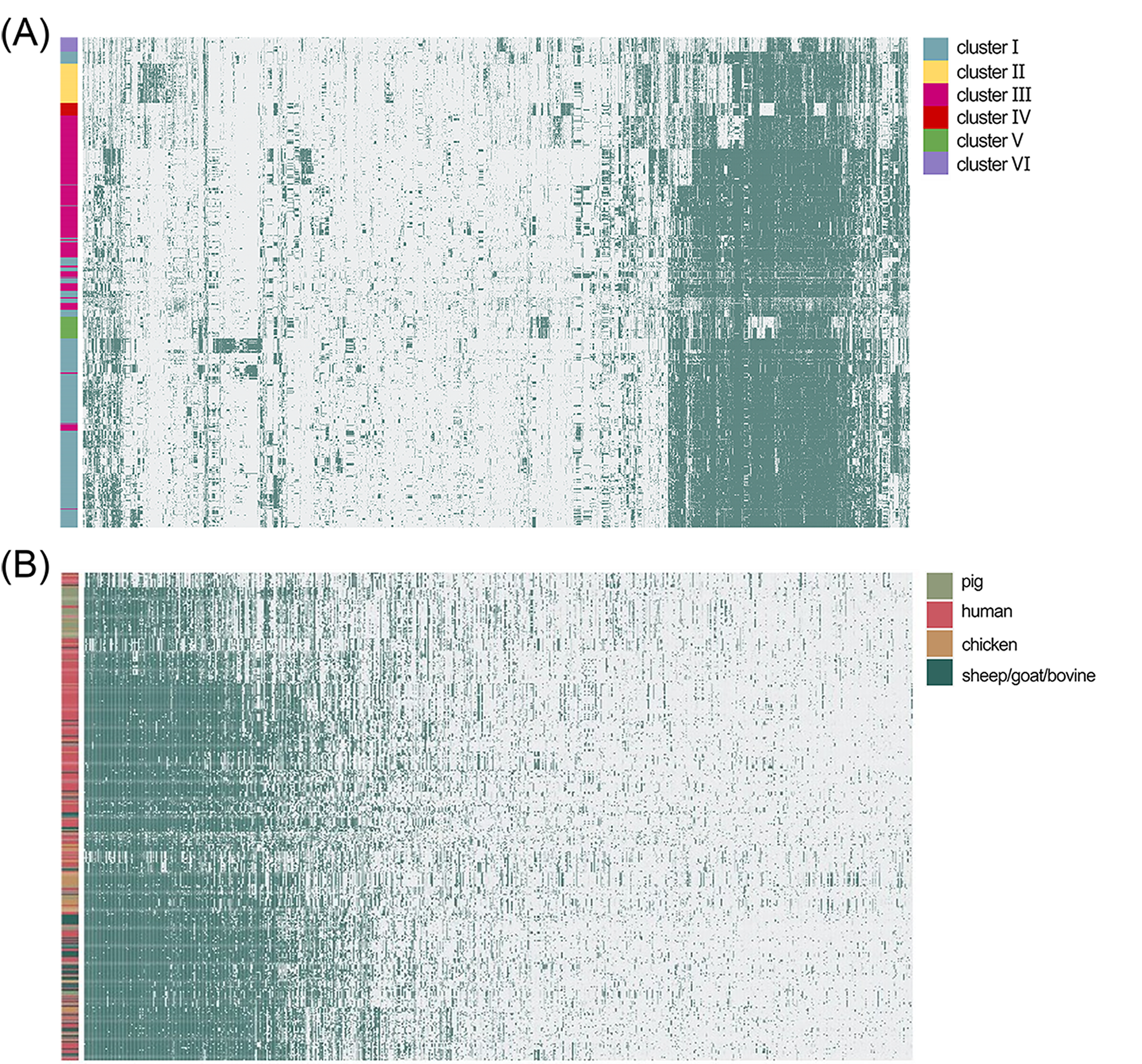
**

**Figure S7** **Comparison of accessory gene profiles of *C. perfringens* strains across different phylogenetic clusters and sources.** (A) Cluster heatmap of the accessory genes (present in 5%-95% of the strains) of different evolutionary clusters. (B) Cluster heatmap of the accessory genes of *C. perfringens* strains from different sources.

**
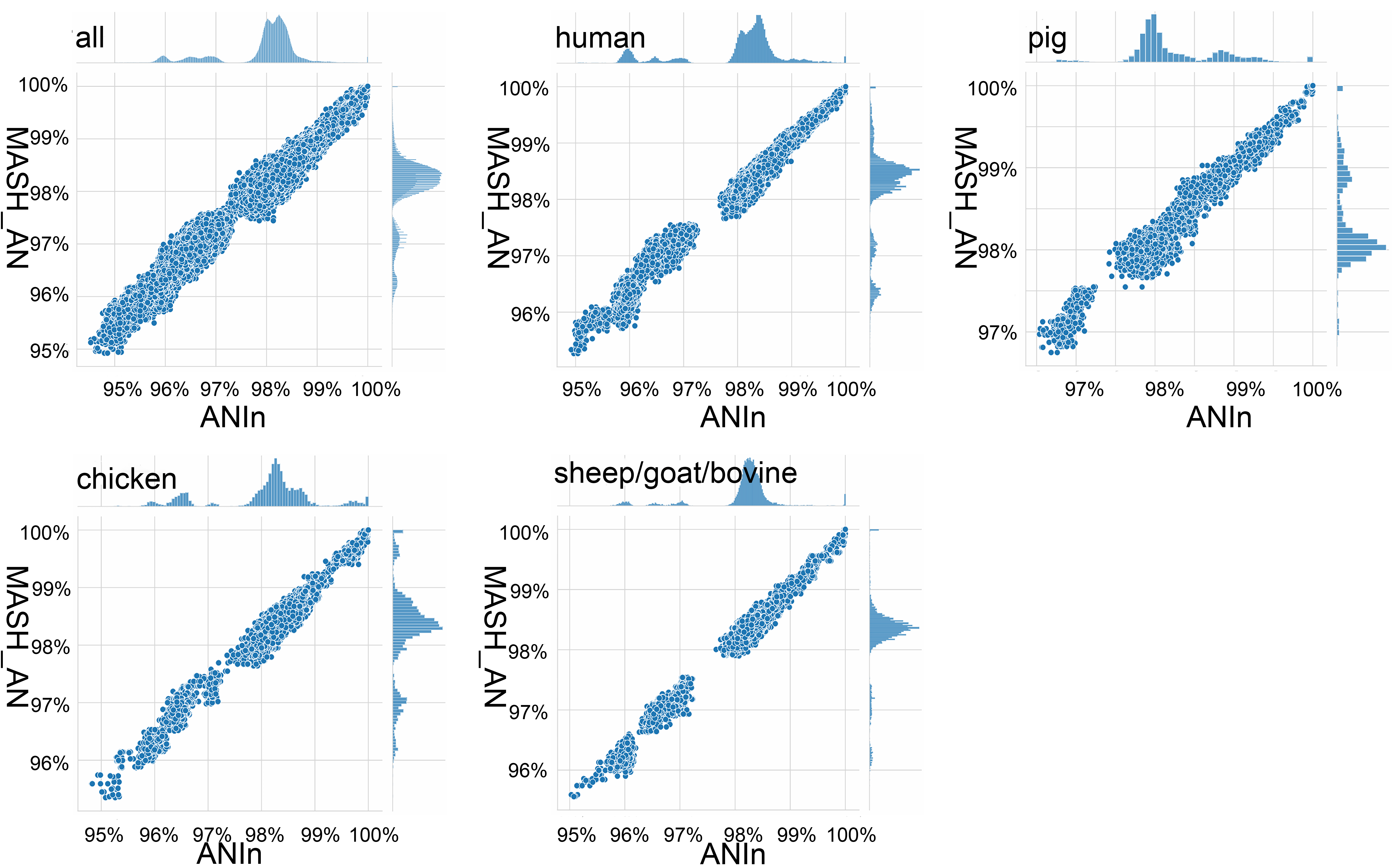
 Figure S8** Average nucleotide identity (ANI) of *C. perfringens* strains from different sources. The X-axis represents the ANI calculated using BLASTn, and the Y-axis represents the ANI calculated using Mash.
